# Traditional Chinese Medicine-derived berberine-loaded herbzyme ameliorates ulcerative colitis by regulating IL10 and TMAO

**DOI:** 10.1016/j.mtbio.2026.103615

**Published:** 2026-08-27

**Authors:** Tao Luo, Meng Li, Ao Zhang, Lu Wang, Qinguo Huang, Zhen Wang, Kang Ding

**Affiliations:** a Nanjing Hospital of Chinese Medicine, Nanjing University of Chinese Medicine, Nanjing, 210000, China; b Department of Neurosurgery, The Second Affiliated Hospital, Shantou University Medical College, Shantou, 515000, China; c Hepatopancreatobiliary Surgery, Xuzhou Central Hospital, Southeast University, Xuzhou, 221000, China

**Keywords:** Carbon dots, Nanozyme, ROS, Ulcerative colitis, BBR, TCM

## Abstract

Ulcerative colitis (UC) is a common inflammatory bowel disease. The traditional Chinese medicine (TCM) formula Zanglian Wan has demonstrated promising therapeutic effects in clinical practice, yet the synergistic mechanisms among its active components remain to be fully elucidated. Inspired by this formula, this study successfully constructed a novel nanocomplex, BLCDs, through the self-assembly of *Sanguisorba officinalis L.*-derived carbon dots (LCDs) and berberine (BBR) via electrostatic interactions and hydrogen bonding. Characterization results showed that LCDs possessed a uniform size distribution and were rich in oxygen-containing functional groups (carboxyl, hydroxyl, carbonyl) on their surface. Their SOD-like activity was dependent on these groups and was retained after BBR conjugation. In LPS-stimulated NCM460 cells, RNA-seq identified IL10 as the most significantly upregulated gene upon BLCDs treatment. Mechanistically, BLCDs promoted the phosphorylation of STAT3 (p-STAT3), which transcriptionally upregulated IL10 expression, leading to inhibition of the NLRP3 inflammasome pathway and its downstream inflammatory cytokines. In DSS-induced mouse model, BLCDs significantly ameliorated intestinal inflammation, oxidative stress, and barrier dysfunction. This therapeutic advantage was attributed to enhanced intestinal retention conferred by their larger particle size and charge switching properties. 16S rDNA sequencing indicated that BLCDs increased gut microbiota abundance and diversity, suppressed the growth of harmful bacteria such as *Klebsiella*, and reduced the generation of trimethylamine N-oxide (TMAO). Colorectal normal organoids (CNOs) experiments further confirmed that BLCDs reversed TMAO-induced inflammatory lesions through an IL10-dependent mechanism. Both *in vitro* and *in vivo* safety evaluations demonstrated that BLCDs possess good biocompatibility. Taken together, this study successfully constructed BLCDs nanocomplexes integrating the antioxidant activity of LCDs and the anti-inflammatory properties of BBR. By modulating p-STAT3-IL10-NLRP3 pathway and the *Klebsiella*-TMAO axis to ameliorate UC, this work establishes a foundation for the development of nanomedicines based on active TCM components.

## Highlights

• Developed a TCM-inspired nano-assemblies (BLCDs) integrating herbal carbon dots and berberine.
• BLCDs promote STAT3 phosphorylation to upregulate IL10 thus inhibiting the NLRP3 inflammasome.
• Demonstrated superior efficacy to 5-ASA in alleviating DSS-induced colitis in mice.
• Revealed novel mechanism: suppressing *Klebsiella* to reduce TMAO, activating IL10 network.
• Confirmed good biocompatibility, highlighting potential for clinical translation.

## Graphical abstract

**Figure:**
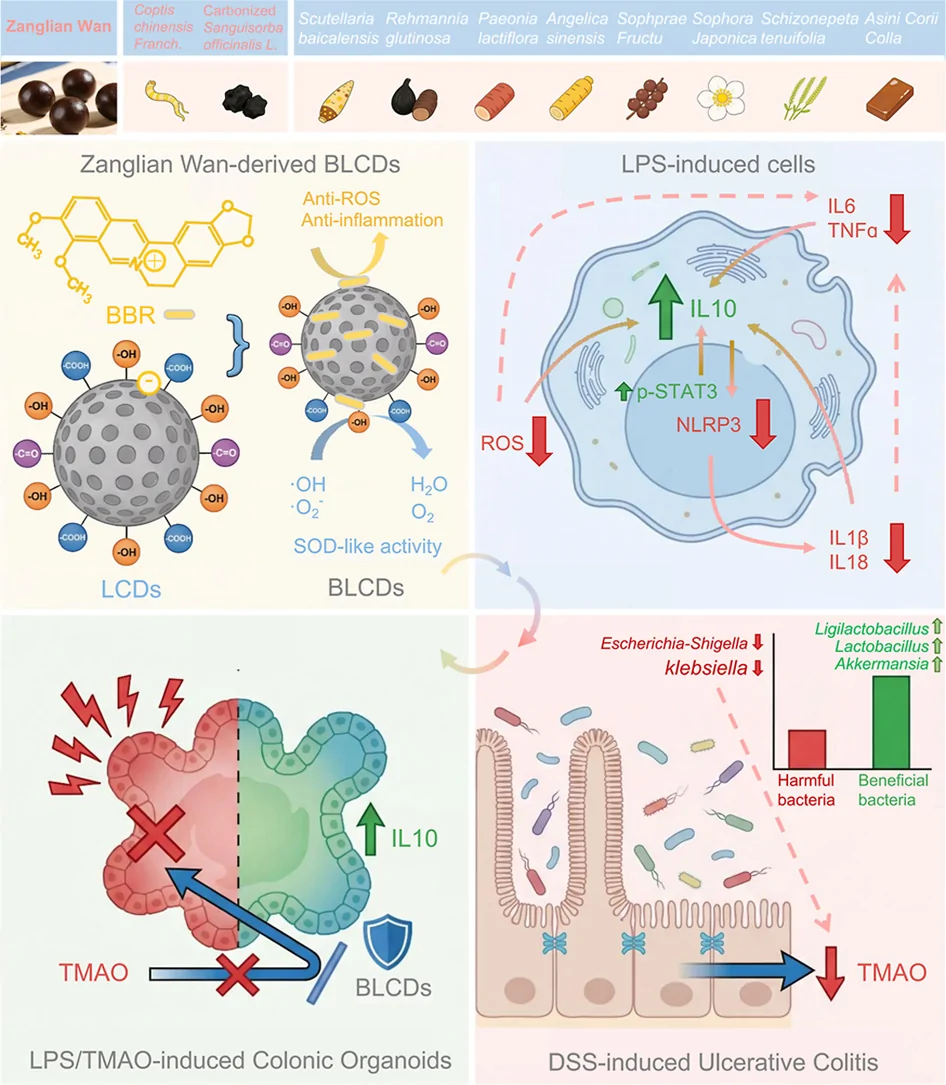

## List of abbreviations

Ulcerative colitis: UC
traditional Chinese medicine: TCM
*Sanguisorba officinalis L.*-derived carbon dots: LCDs
Berberine: BBR
trimethylamine N-oxide: TMAO
Colorectal normal organoids: CNOs
Mesalazine: 5-ASA
Trimethylamine: TMA

## 1 Introduction

Ulcerative colitis (UC) is a chronic inflammatory disease of the colon and rectum, primarily affecting the mucosa and submucosa, with an unknown etiology [1]. Its clinical course is marked by recurrent episodes and long-term persistence, which has led to its characterization as a “green cancer.” The pathogenesis of UC is complex and multifactorial, involving genetic susceptibility, immune dysregulation, alterations in the gut microbiota, and environmental triggers [[2], [3], [4]]. At present, no curative treatment is available, and the disease is associated with an increased risk of colorectal cancer over time [5]. In recent years, driven by lifestyle changes and improved diagnostic techniques, the global burden of UC has risen steadily, establishing it as a growing public health concern [6].

Zanglian Wan, a traditional Chinese medicinal formula documented in the Chinese Pharmacopoeia, is primarily indicated for intestinal inflammation and stopping bleeding [7]. It comprises ten herbal components, including *Coptis chinensis Franch.*, carbonized *Sanguisorba officinalis L.*, *Scutellaria baicalensis*, *Rehmannia glutinosa, Paeonia lactiflora*, *Angelica sinensis*, *Sophprae Fructus*, *Sophora Japonica*, *Schizonepeta tenuifolia*, and *Asini Corii Colla*. Among these, *Coptis chinensis Franch.* and carbonized *Sanguisorba officinalis L.* function as the sovereign and minister herbs, respectively, and are considered pharmacologically critical. However, the specific mechanism underlying their synergistic interaction remains to be fully elucidated.

Berberine (BBR, C_20_H_18_NO_4_^+^), the principal active constituent of *Coptis chinensis Franch.*, carries a positive charge due to a quaternary ammonium nitrogen atom in its structure [8]. BBR has attracted considerable attention for its multi-target pharmacological effects in the treatment of UC [9]. Accumulating evidence indicates that BBR ameliorates colitis through multiple mechanisms, including the regulation of pro-inflammatory cytokine expression [10], enhancement of intestinal epithelial barrier function via upregulation of tight junction proteins [11], modulation of gut microbiota composition [12], and intervention in key signaling pathways such as NF-κB, NRF2, EIF2AK2, JAK-STAT, AKT, and NLRP3 [[13], [14], [15], [16], [17], [18]]. In addition, BBR alleviates oxidative stress damage in colonic tissue and suppresses the release of inflammatory mediators [19]. Despite these promising pharmacological properties, the clinical application of BBR is hindered by several challenges, including poor water solubility, low oral bioavailability, and insufficient accumulation at sites of colonic inflammation [20]. Therefore, the development of novel formulation strategies to overcome these limitations is essential for advancing the clinical translation of BBR.

Traditional Chinese Medicine (TCM) represents a rich reservoir of pharmacological resources. Among these, carbonized TCM, a unique class of processed herbal products, have been utilized clinically for over a millennium due to their traditional efficacy in anti-inflammation, hemostasis, and anti-diarrhea [21]. Recent studies have revealed that TCM-derived carbon dots (TCM-CDs) are key active herbzyme generated during the carbonization process [[21], [22], [23]]. Synthesized through high-temperature carbonization of herbs, these TCM-CDs exhibit exhibit enzyme-like activities analogous to those of natural oxidase (OXD), glucose oxidase (GOD), peroxidase (POD), catalase (CAT), and superoxide dismutase (SOD) [24,25]. Notably, TCM-CDs not only efficiently scavenge reactive oxygen species (ROS) but also possess good biocompatibility and *in vivo* safety [3], demonstrating significant potential value in antioxidant and anti-inflammatory clinical applications.

The self-assembly of active molecules in TCM formulas represents an emerging frontier in the modernization of TCM research [26]. This process involves the spontaneous formation of structurally stable, ordered nano-aggregates with specific functions, driven by weak intermolecular interactions such as non-covalent bonds among active ingredients, including flavonoids, alkaloids, and saponins [[26], [27], [28]]. These assemblies can occur during decoction or after entering the organism. To date, most studies have been primarily limited to the self-assembly of active small molecules [29], while the interactions and synergistic mechanisms between nanomaterials such as TCM-CDs and active small molecules remain largely unexplored and warrant further investigation.

In this study, drawing inspiration from Zanglian Wan, we synthesized carbon dots derived from *Sanguisorba officinalis L.*-derived carbon dots (LCDs) via a one-step hydrothermal method. These LCDs were subsequently assembled with BBR through electrostatic interactions and hydrogen bonding to form a novel nanomedicine, designated BLCDs. This work systematically investigated the SOD-like activity and ROS scavenging mechanism of LCDs, and explored the synergistic anti-inflammatory effects resulting from the assembly of BBR and LCDs. Utilizing RNA-seq, 16S rDNA analysis, untargeted metabolomics, as well as cellular, animal, and colonic organoid (CNOs) models, we systematically elucidated the mechanism by which BLCDs, derived from Zanglian Wan, ameliorate UC through the regulation of IL10 and TMAO.

## 2 Materials and methods

### 2.1 Synthesis of LCDs and BLCDs

*Sanguisorba officinalis L.* was obtained from Nanjing Hospital of Chinese Medicine and pulverized into a fine powder using an herbal grinder. The powder was then mixed with ddH_2_O at a ratio of 1:10 (w/v) in a reactor and heated at 200°C for 8 h. After the reaction, the product was filtered, dialyzed for 24 h, and subjected to vacuum freeze-drying. The resulting product was designated LCDs and stored at 4°C for long-term preservation. The prepared LCDs were mixed with BBR at a mass ratio of 1:2, subsequently dissolved in ddH_2_O at a ratio of 1:10 (w/v), and transferred to a reactor for hydrothermal reaction. After heating at 100°C for 1 h, the reaction product was filtered, dialyzed for 24 h, and vacuum freeze-dried. The final product was designated BLCDs and stored at 4°C for long-term preservation. The morphology, particle size, and zeta potential of LCDs and BLCDs were characterized using transmission electron microscopy (TEM, FEI Tecnai F20) and a nanoparticle size and zeta potential analyzer (Malvern Zetasizer Nano ZS90). Spectral characterization was performed using Fourier transform infrared spectroscopy (FT-IR, Nexus 670), ultraviolet-visible spectrophotometry (UV-Vis, PE Lambda 950), and fluorescence spectrometry (FLS 980). Elemental and chemical state analyses were conducted via X-ray photoelectron spectroscopy (XPS, Thermo, Kalpha). Additionally, the structural properties of LCDs were further analyzed using Raman spectroscopy (Raman, Thermo, DXR) and X-ray diffraction (XRD, D8).

### 2.2 ROS detection

For the ABTS assay, an ABTS working solution was prepared by mixing ABTS solution (7.4 mmol/L) with K_2_S_2_O_8_ solution (2.6 mmol/L) and allowing the mixture to stand in the dark at room temperature for 16 h. The ABTS working solution was then diluted with PBS to achieve an appropriate absorbance at 734 nm. Subsequently, 0.2 mL of the diluted ABTS working solution was mixed with 10 μL of LCDs or BLCDs solution (final concentrations of 0, 10, 25, 50, and 100 μg/mL). The mixture was placed in a microplate reader (BioTek, Winooski, VT, USA), and the absorbance at 734 nm was measured at 6 min to evaluate the ABTS radical scavenging activity of the samples.

The salicylic acid (SA) method is based on the Fenton reaction system, in which hydroxyl radicals (•OH) react with SA to generate 2,3-dihydroxybenzoic acid (2,3-DHBA), which exhibits characteristic UV absorption at 510 nm. Upon the addition of LCDs or BLCDs (final concentration: 0, 10, 25, 50, and 100 μg/mL) with •OH scavenging activity, the •OH radicals in the system decreased, resulting in a corresponding reduction in absorbance at 510 nm. The reaction mixture (containing SA, FeSO_4_, LCDs/BLCDs, and H_2_O_2_) was diluted with PBS, incubated at 37°C for 20 min, and then the absorbance at 510 nm was measured using a UV-Vis spectrophotometer. The mean absorbance ± standard deviation and the •OH scavenging rate of LCDs or BLCDs were calculated, and a sample concentration-scavenging rate curve was plotted to evaluate their dose-dependent scavenging effects. Trolox was used as a positive control.

In the pyrogallol assay, 10 μL of LCDs or BLCDs solution was added to 2940 μL of Tris-HCl buffer (0.05 mol/L, pH 7.4) containing Na_2_EDTA (1 mmol/L). Subsequently, 50 μL of pyrogallol solution (prepared in 1 mmol/L HCl at a concentration of 60 mmol/L) was added, and the mixture was immediately mixed rapidly by shaking at room temperature. The absorbance of the mixture at 325 nm was measured at 3 min using a spectrophotometer (FLS 980). The superoxide anion radical (•O_2_^−^) scavenging rate was calculated using the following formula: (A – A_0_)/A_0_ × 100%, where A_0_ represents the increase in absorbance of the mixture at 325 nm without the sample, and A represents the corresponding increase in absorbance after the addition of the sample. Trolox was used as a positive control.

The SOD-like activities of the carbon dots were assessed with a Total Superoxide Dismutase Assay Kit (S311-10, Dojindo Molecular Technologies) in accordance with the manufacturer's protocol.

For the DCFH staining assay, NCM460 cells were seeded in 6-well plates and cultured for 24 h. The cells were then treated with LPS (20 μg/mL) for 24 h. After induction, the cells were co-incubated with LCDs (16.7 μg/mL), BBR (33.3 μg/mL), or BLCDs (50 μg/mL) for 24 h. Finally, the cells were incubated with 10 μmol/L DCFH-DA working solution in the dark at 37°C with 5% CO_2_ for 30 min. After washing away excess dye with PBS, the intracellular ROS level was observed using a fluorescence microscope and quantitatively analyzed using a microplate reader.

### 2.3 Cell viability

The cell lines used in this study were obtained from the Cell Resource Center of the Shanghai Institute for Biological Sciences, Chinese Academy of Sciences, including NCM460 (human normal intestinal epithelial cells) and RAW264.7 (mouse mononuclear macrophage leukemia cells). RAW264.7 cells were cultured in DMEM medium, while NCM460 cells were maintained in 1640 medium. All culture media were supplemented with 10% fetal bovine serum (HyClone), 100 U/mL penicillin (Thermo), and 100 μg/mL streptomycin (Thermo). Cells were incubated at 37°C in a humidified atmosphere containing 5% CO_2_. Cell viability was assessed using the CCK-8 assay kit (Yeasen). NCM460 cells were seeded into 96-well plates at a density of 5 × 10^3^ cells per well and cultured overnight. Subsequently, the cells were stimulated with LPS (20 μg/mL) for 24 h. After induction, the cells were treated with LCDs (16.7 μg/mL), BBR (33.3 μg/mL), or BLCDs (50 μg/mL) for 24 h. Following incubation, 10 μL of CCK-8 reagent was added to each well, and the plates were incubated for an additional 1 h. The absorbance at 450 nm was measured using a microplate reader.

### 2.4 AO/EB staining

Cells in the logarithmic growth phase were seeded into 24-well plates at a density of 1 × 10^5^ cells per well and cultured overnight. Subsequently, the cells were stimulated with LPS (20 μg/mL) for 24 h. After treatment, the cells were incubated with LCDs (16.7 μg/mL), BBR (33.3 μg/mL), or BLCDs (50 μg/mL) for 48 h. Following incubation, all cells were stained with AO/EB staining solution (Sangon, E607308) according to the manufacturer's instructions. Briefly, the culture medium was aspirated, and PBS buffer was added. The staining solution was prepared by adding 10 μL of AO and 10 μL of EB per 180 μL of PBS buffer, and cells were incubated with the mixture in the dark at room temperature for 5 min. Viable cells appeared uniformly green, while necrotic cells were stained orange. Cell imaging was performed using a fluorescence microscope to observe live and dead cells.

### 2.5 RNA-seq analysis

RNA sequencing (RNA-Seq) was performed by MAGIGENE (Shenzhen, China) with three biological replicates per treatment group. A total of 12 RNA samples were subjected to sequencing analysis, including four independently extracted RNA samples from NCM460 cells: PBS group, BLCDs group, LPS-induced group before and after BLCDs treatment. After obtaining transcript read counts, the R package tximport was used to convert transcript-level read counts to gene-level read counts. Differential expression analysis was subsequently performed using DESeq2 software. Differentially expressed genes (DEGs) were identified based on the criteria of FDR < 0.05 and |fold change| ≥ 1.5. Gene Ontology (GO) and Kyoto Encyclopedia of Genes and Genomes (KEGG) pathway enrichment analyses were conducted on the selected DEGs.

### 2.6 ELISA assay

The following ELISA kits were used for detection: IL6 ELISA kit (Abcam, ab178013 for human, ab222503 for mouse), TNF-α ELISA kit (Abcam, ab181421 for human, ab208349 for mouse), IL1β ELISA kit (Abcam, ab214025 for human, ab197742 for mouse), IL1α ELISA kit (Abcam, ab100560 for human), IL10 ELISA kit (Abcam, ab185986 for human, ab255729 for mouse), NLRP3 ELISA kit (Abcam, ab274401 for human, ab279417 for mouse), MDA ELISA kit (KeyGEN, KGC8405-48), GSH ELISA kit (JINGMEI, JM-5390H1), and IL18 ELISA kit (Abcam, ab215539 for human, ab216165 for mouse). The main procedures were as follows: reagents were prepared according to the manufacturer's instructions and allowed to reach room temperature. Then, 50 μL of sample or standard was added to each well, followed by 50 μL of antibody cocktail. After incubation at room temperature for 1 h, the supernatant was discarded, and the wells were washed three times with Wash Buffer. Subsequently, 100 μL of TMB substrate was added to each well and incubated in the dark for 15 min for color development. The reaction was terminated by adding 100 μL of stop solution, and the optical density (OD) was immediately measured at 450 nm.

### 2.7 RT-qPCR

Cells (1 × 10^7^) were lysed using 1 mL of TRIzol (Sigma), and 200 μL of chloroform was added to extract the aqueous phase containing RNA. RNA was precipitated by adding 1 mL of isopropanol, followed by washing with 200 μL of 75% ethanol. The RNA pellet was then dissolved in 100 μL of ddH_2_O, and the total RNA concentration was determined using a Nanodrop spectrophotometer. All procedures were performed under RNase-free conditions to prevent RNA degradation. Complementary DNA (cDNA) was synthesized by reverse transcription using the HiScript II 1st Strand cDNA Synthesis Kit (Vazyme), followed by real-time qPCR with the HiScript II One Step RT-PCR Kit (Vazyme). GAPDH was used as the reference gene, and the relative mRNA transcription levels were calculated using the 2^−ΔΔCt^ method. Primer information is provided in Table S1. All experiments were performed in triplicate to ensure reliability of the results.

### 2.8 Treatment of ulcerative colitis

In the UC model experiments, 30.0 g of dextran sulfate sodium (DSS) was dissolved in 1 L of deionized water (DI water) to prepare a 3% DSS solution. Eight-week-old female BALB/c mice were randomly divided into seven groups (n = 6): PBS group (healthy control), DSS group (model group), 5-ASA group (mesalazine, positive control), LCDs group, BBR group, BBR + LCDs group (mixture of BBR and LCDs), and BLCDs group. Body weight and fecal conditions of the mice were observed and recorded throughout the experiment. After 7 days of adaptive feeding, modeling was initiated. From day 0 of modeling, mice in the DSS group and all treatment groups were given free access to 3% DSS solution. Body weight and fecal conditions were continuously monitored for 14 days. Drug administration was initiated following induction of the disease model. The 5-ASA group received 5-ASA at 100 mg/kg/day via oral gavage daily for 7 consecutive days. The other treatment groups, including LCDs (26.67 mg/kg), BBR (53.33 mg/kg), BBR + LCDs (the physical mixture of 53.33 mg/kg BBR and 26.67 mg/kg LCDs), and BLCDs (80 mg/kg), were orally administered on days 1, 3, and 5 (every other day) over the same 7-day period. The Disease Activity Index (DAI), which integrates scores for body weight loss, stool consistency, and fecal occult blood, was used to assess the degree of inflammation in each group. Anal photographs were taken before and after treatment. At the end of the treatment period, the mice were euthanized, and the colons were excised and measured for length. Colon tissue sections were subjected to H&E staining, Alcian Blue (AB) staining, and Picro-Sirius Red (PSR) staining.

In addition, Cy5.5 fluorescent dye was conjugated to LCDs and BLCDs using the EDC/NHS method. After dialysis and vacuum drying, the fluorescently labeled products were obtained. A standard curve was established to correlate the fluorescence intensity of Cy5.5 with the concentration of LCDs or BLCDs. Subsequently, DSS-induced mice were administered LCDs or BLCDs (80 mg/kg, p.o.). Animals were euthanized at 0, 1, 3, 6, 12, and 24 h post-administration, and major tissues (heart, liver, spleen, lungs, kidneys, and colon) were dissected for near-infrared fluorescence imaging. The biodistribution of LCDs and BLCDs was also assessed at 24, 48, and 72 h post-administration, with sampled tissues including heart, liver, spleen, lungs, kidneys, colon, feces, blood, and urine. The concentrations of LCDs or BLCDs in tissues were calculated based on the standard curve of Cy5.5 fluorescence intensity versus sample concentration, and the results were expressed as the percentage of injected dose per gram of tissue (% ID/g).

### 2.9 Conjugation of Cy5.5 to LCDs and BLCDs

The fluorescent labeling of LCDs or BLCDs with Cy5.5 was achieved via standard EDC/NHS coupling chemistry. Briefly, 10 mg of carboxyl-rich CDs were dispersed in 5 mL of MES buffer (0.1 M, pH 6.0). Subsequently, EDC (10 mg) and NHS (10 mg) were added to the solution, and the mixture was stirred at room temperature for 30 min to activate the carboxyl groups on the surface of the LCDs or BLCDs. After activation, the pH of the solution was adjusted to 7.4 using a PBS buffer (10X) or dilute NaOH. Then, 1 mg of amine-functionalized Cy5.5 (Cy5.5-NH_2_) dissolved in a minimal amount of DMSO was added dropwise to the activated LCDs or BLCDs solution. The reaction mixture was continuously stirred for 12 h at room temperature in the dark. To remove unreacted Cy5.5, EDC, NHS, and other byproducts, the resulting solution was transferred into a dialysis bag and dialyzed against deionized water for 48 h in the dark. The dialysate was replaced every 6 h until no fluorescence was detected in the external water. Finally, the purified Cy5.5-labeled LCDs or BLCDs (Cy5.5-LCDs or BLCDs) were lyophilized and stored at 4°C in the dark for further use.

### 2.10 H&E, AB, and PSR staining assays

H&E staining was performed on heart, liver, spleen, lung, kidney, and colon tissues. Paraffin sections were deparaffinized and rehydrated, followed by staining with hematoxylin for 5 min. The sections were then differentiated in hydrochloric acid ethanol, blued in ammonia water, and counter stained with eosin for 3 min. After dehydration through a graded ethanol series, the sections were cleared in xylene and mounted with neutral balsam. Under light microscopy, nuclei appeared blue-purple, while the cytoplasm and extracellular matrix appeared pink.

AB staining was performed on colon tissue sections. Following deparaffinization and rehydration, the sections were stained with AB solution (pH 2.5) for 30 min, then rinsed with running water. Nuclei were lightly counterstained with nuclear fast red. After dehydration, clearing, and mounting, acidic mucopolysaccharides appeared blue, and nuclei appeared red under light microscopy.

PSR staining was also performed on colon tissue sections. All reagents and materials were allowed to return to room temperature. After deparaffinization and rehydration, the sections were incubated with PSR staining solution for 60 min, rapidly rinsed twice with acetic acid solution, dehydrated with absolute ethanol, cleared in xylene, and mounted with synthetic resin. PSR specifically binds to collagen fibers, appearing red under light microscopy. The extent of colonic tissue fibrosis was quantitatively assessed by calculating the percentage of red-positive area using ImageJ software based on color threshold analysis.

### 2.11 Immunofluorescence assay

After fixation, colon tissue samples were incubated with primary antibodies against ZO-1 (Abcam, ab307799, 1:500) and Occludin (Abcam, ab216327, 1:200) overnight at 4°C. Following three washes with TBST, the sections were incubated in the dark with Alexa Fluor 647-conjugated secondary antibody (Abcam, ab300101, 1:100) for ZO-1 detection or Alexa Fluor 488-conjugated secondary antibody (Abcam, ab150077, 1:500) for Occludin detection. After incubation, the sections were washed three times with TBST (5 min each), followed by DAPI staining (Beyotime, C1005). Following a 5-min incubation at room temperature, the staining solution was removed, and the sections were washed three times with TBST (5 min each). The stained sections were then directly observed under a fluorescence microscope.

### 2.12 Colorectal normal organoids culture

Patient-derived colorectal normal organoids (CNOs) were recovered from cryopreserved organoid lines. The organoids were resuspended in Matrigel and seeded into 6-well plates. Culture was performed using a human colon organoid culture kit (MCE, HY-K6112), with the culture medium replaced every 48 h. Passaging was conducted every 7-10 days by mechanical pipetting using a pipette tip or, when necessary, by digestion with 1-2 mL of TrypLE™ Express (GIBCO). For CNO experiments, the organoids were first treated with LPS (50 μg/mL) or TMAO (200 μM) for 24 h. After induction, BLCDs (50 μg/mL) were added to the designated wells and incubated for 48 h. Finally, changes in CNO diameter were observed under a microscope. Cell viability was assessed using the CCK-8 assay, and MDA levels were detected by ELISA.

### 2.13 Immunohistochemistry staining assay

Immunohistochemical staining for IL10 and NLRP3 was performed on colon tissue or CNO sections. After deparaffinization and rehydration, antigen retrieval was conducted by incubating the sections in citrate buffer (pH 6.0). Endogenous peroxidase activity was blocked with 3% H_2_O_2_, and non-specific binding sites were blocked with 5% BSA. Subsequently, the sections were incubated overnight at 4°C with primary antibodies against IL10 (Abcam, ab133575 for human, ab9969 for mouse, 1:200) or NLRP3 (Invitrogen, MA5-23919, 1:100). Following washing with PBS, HRP-conjugated goat anti-rabbit IgG secondary antibody (Abcam, ab288151, 1:5000) was applied and incubated at room temperature for 1 h. After another washing step, DAB substrate was used for color development, and the sections were counterstained with hematoxylin for nuclear visualization, then mounted. Positive signals (brown precipitates) were observed under a light microscope.

### 2.14 16S rDNA sequencing analysis

Genomic DNA was extracted from fecal samples of mice in each group (PBS, DSS, 5-ASA, LCDs, BBR, BBR + LCDs, and BLCDs; n = 6 per group). The extracted DNA was required to meet the following quality criteria: concentration >50 ng/μL, total amount >5 μg, OD260/280 ratio between 1.8 and 2.0, and no obvious RNA bands detected by electrophoresis. Library construction was performed following the standard protocol of the NEBNext Ultra™ DNA Library Prep Kit for Illumina, and high-throughput sequencing was conducted on the HiSeq platform. Raw data were subjected to quality control filtering to remove low-quality reads, and the resulting clean data were used for subsequent analysis. Paired-end reads were merged into tags, and redundant sequences were removed to obtain unique tags. The unique tags were clustered into operational taxonomic units (OTUs). Based on OTU annotation, species classification and abundance statistics were performed. Subsequent analysis covered the characterization of microbial community composition, diversity, and statistically significant differences between groups.

### 2.15 Untargeted metabolomics sequencing and analysis

Metabolomics analysis was performed by MAGIGENE (Shenzhen, China) on DSS-induced mouse colon tissue samples before and after BLCDs treatment, with six biological replicates per group (designated as DSS group and BLCDs group). Untargeted metabolomics analysis was conducted using LC-MS/MS technology. Samples were analyzed using a Vanquish ultra-high performance liquid chromatography system coupled with a Q-Exactive high-resolution mass spectrometer (Thermo), with chromatographic separation performed on a Hypersil Gold column (100 × 2.1 mm, 1.9 μm, Agilent). Raw data were processed using Compound Discoverer 3.3 software (Thermo) and matched against the mzCloud, mzVault, and MassList databases for relative quantification of metabolites. Orthogonal partial least squares discriminant analysis (OPLS-DA) and differential metabolite screening were subsequently performed on the dataset.

### 2.16 *In vivo* safety tests

For the safety assessment study, eight-week-old female BALB/c mice were randomly divided into two groups (PBS group and BLCDs group, n = 10). Mice were administered PBS (100 μL) or BLCDs (80 mg/kg) (p.o.), and body weight changes were monitored daily. On day 7, mice were euthanized, and whole blood and serum samples were collected for hematological and biochemical analyses of liver and kidney function. Meanwhile, heart, liver, spleen, lung, and kidney tissues were harvested for H&E staining. Whole blood samples were analyzed using a Hematology Analyzer (Mindray, BC-60R) to determine red blood cell (RBC), white blood cell (WBC), platelet (PLT), and hemoglobin (HGB) levels. Serum samples were prepared by allowing whole blood to stand at room temperature for 2 h, followed by centrifugation at 4°C and 3000 rpm for 15 min. The supernatant was immediately analyzed using an Automatic Biochemical Analyzer (BIOBASE, BK-400).

Hemocompatibility of BLCDs was evaluated using 1 mL of mouse blood. Red blood cells were isolated by repeated centrifugation. Then, 50 μL of the erythrocyte suspension was mixed with 450 μL of BLCDs at various concentrations and incubated at 37°C for 3 h. Erythrocytes treated with PBS and ddH_2_O served as negative and positive controls, respectively. Following incubation, the mixtures were centrifuged, and the absorbance of the supernatant was measured at 570 nm to calculate the hemolysis rate.

### 2.17 Statistical analysis

All experimental data were expressed as mean ± standard deviation (Mean ± SD). Statistical analysis and graph generation were performed using GraphPad Prism 8 software. Comparisons between two groups were conducted using a two-tailed Student's t-test. For comparisons involving three or more groups, one-way or two-way analysis of variance (ANOVA) was employed, followed by Tukey's or Sidak's multiple comparisons test as appropriate. A p value < 0.05 was considered statistically significant (*p < 0.05, **p < 0.01, ***p < 0.001, ****p < 0.0001).

## 3 Results

### 3.1 Preparation and characterization of BLCDs

**Fig. 1:**
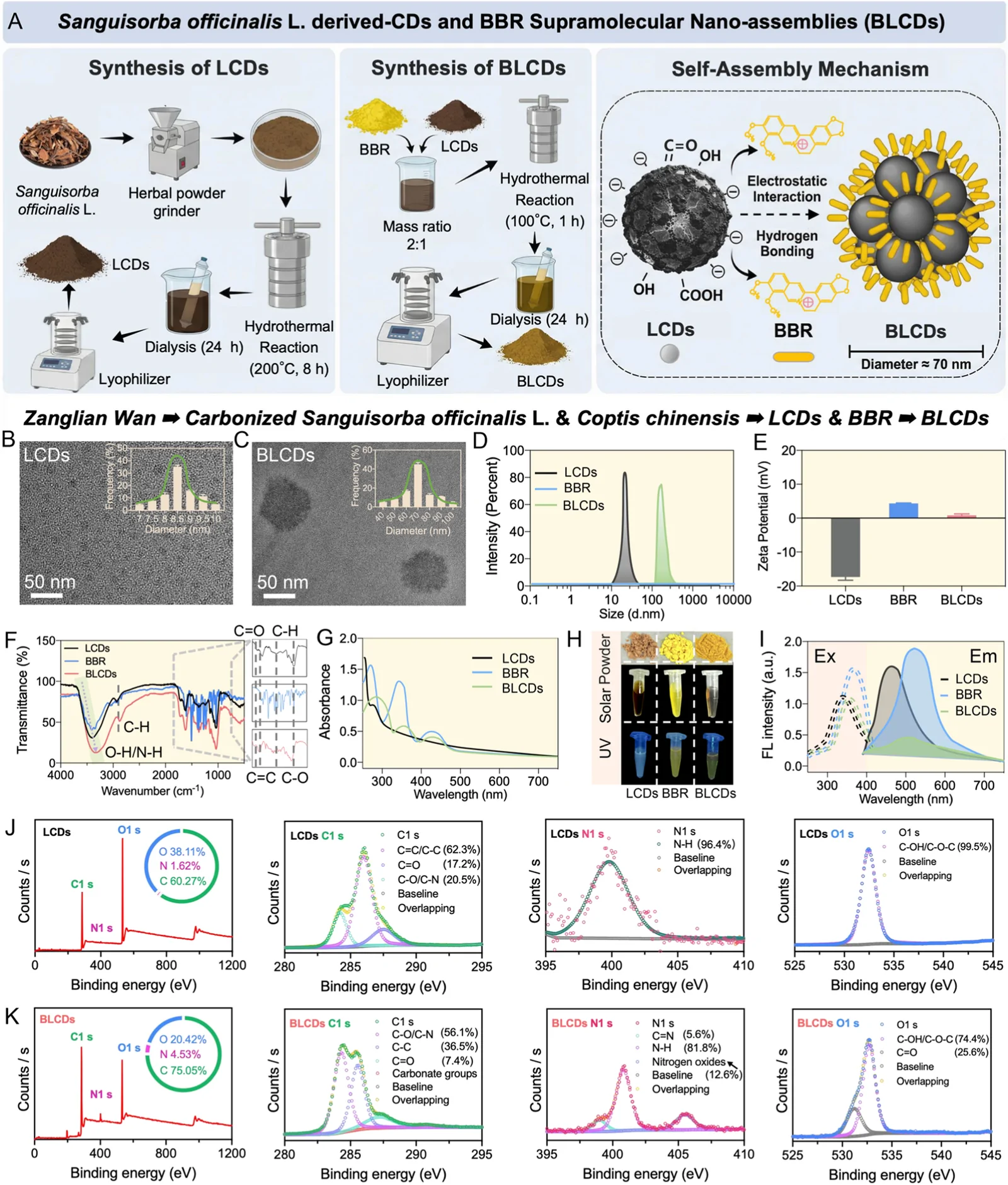
**Synthesis and characterization of BLCDs.** (A) Schematic illustration of BLCDs synthesis. (B) TEM image of LCDs; (C) TEM image of BLCDs; inset showed the particle size distribution. (D) Hydrodynamic size distribution of LCDs, BBR, and BLCDs. (E) Zeta potential of LCDs, BBR, and BLCDs (n = 3). (F) FT-IR spectra of LCDs, BBR, and BLCDs. (G) UV-vis absorption spectra of LCDs, BBR, and BLCDs. (H) Photographs of LCDs, BBR, and BLCDs powders and their aqueous solutions under sunlight and UV light. (I) Excitation and emission spectra of LCDs, BBR, and BLCDs. (J) XPS survey spectrum and high-resolution C 1s, N 1s, and O 1s spectra of LCDs. (K) XPS survey spectrum and high-resolution C 1s, N 1s, and O 1s spectra of BLCDs.

We pulverized *Sanguisorba officinalis L.* using an herbal powder grinder, followed by a one-step hydrothermal reaction in a reactor at 200°C for 8 h. The product was dialyzed and vacuum freeze-dried to obtain carbon dots derived from *Sanguisorba officinalis L.*, designated as LCDs (Fig. 1A). TEM results showed that the synthesized LCDs were uniform in size without obvious aggregation, exhibiting a Gaussian particle size distribution with an average diameter of 8.5 ± 0.35 nm (Fig. 1B). High-resolution transmission electron microscopy (HRTEM) imaging revealed that the characteristic lattice fringes of the LCDs exhibited a spacing of approximately 0.32 nm (Fig. S1A), which can be assigned to the (002) plane of graphitic carbon [30], indicating the presence of locally ordered graphitic nanodomains within the carbon core. Further selected area electron diffraction (SAED) pattern revealed that LCDs possessed a diffuse ring pattern [25], indicating a lack of long-range order and suggesting an amorphous carbon structure in the bulk of the LCDs (Fig. S2A). Concurrently, XRD data showed a broad diffraction peak for LCDs at 24.5° (Fig. S2B), corresponding to the (002) plane of graphene, indicating a highly disordered graphitic structure [31].

To elucidate the structure and composition of LCDs, we performed preliminary characterization using Raman spectroscopy. The data revealed characteristic Raman peaks for LCDs, namely the D-band at 1380 cm^−1^ and the G-band at 1576 cm^−1^ (Fig. S2C), confirming the formation of carbon dots. The G peak corresponded to the in-plane vibration of sp^2^ carbon atoms, whereas the D peak was a disorder-induced breathing mode, serving as an indicator of defect density [32]. The intensity ratio of the D-band to G-band (I_D_/I_G_) was 0.74, indicating a graphene-like structure for LCDs. To quantitatively analyze the relative content of sp^2^ and sp^3^ hybridized carbon, we performed baseline correction on the Raman spectrum and conducted peak fitting for the D and G bands. In the deconvoluted spectrum, peaks at 1350 cm^−1^ and 1605 cm^−1^ were attributed to sp^2^ carbon, while peaks at 1195 cm^−1^ and 1510 cm^−1^ were attributed to sp^3^ carbon. The integrated intensity ratio of sp^2^ to sp^3^ carbon was approximately 2.59, yielding a calculated relative content of 72.2% for sp^2^ carbon and 27.8% for sp^3^ carbon in LCDs. These results indicated that LCDs possessed a graphitization degree of 72.2% while also containing abundant amorphous carbon.

Inspired by the preparation process of Zanglian Wan, which involves pulverizing herbs like *Coptis chinensis Franch.* and carbonized *Sanguisorba officinalis L.* followed by heating until thoroughly steamed, we mixed the active component of *Coptis chinensis Franch.*, BBR, with LCDs at different ratios in a reactor for hydrothermal reaction at 100°C for 1 h. The results showed that the loading efficiency of BBR onto LCDs was highest when the BBR to LCDs ratio was 2:1 (Fig. S2D). After dialysis and vacuum freeze-drying, the synthesized product was named BLCDs. TEM results showed that BLCDs were approximately spherical with a diameter of about 72.4 ± 2.1 nm, but with uneven edges and blurred interface contours, suggesting that their surface might be grafted with a large amount of BBR (Fig. 1C). Particle size analysis further revealed that the hydrodynamic diameter of BLCDs in aqueous solution was significantly larger than that of LCDs (Fig. 1D). To further verify the dispersion stability of BLCDs, we measured the hydrodynamic diameters and PDI values in ddH_2_O, PBS, and simulated intestinal fluids (SIF). The results showed slight fluctuations in particle size across different media, with PDI values consistently maintained within 0.15-0.3, and no significant aggregation or dramatic increase in particle size was observed (Fig. S3). These findings indicated that BLCDs possessed good dispersion stability in aqueous solutions. Zeta potential measurements showed that the surface potential of LCDs was −17.8 mV, BBR was +4.1 mV, while BLCDs was +0.82 mV (Fig. 1E). These data indicated that LCDs and BBR could self-assemble through electrostatic interactions to form supramolecular nano-assemblies, namely BLCDs.

Fourier transform infrared spectroscopy (FT-IR) was employed to analyze surface functional groups. The results showed characteristic peaks for LCDs at 3423 cm^−1^ (O-H/N-H), 2751 cm^−1^ (C-H), 1650 cm^−1^ (C=C/C=O), and 1021 cm^−1^ (C-O) (Fig. 1F). However, upon binding with BBR, the O-H/N-H characteristic peak of BLCDs redshifted to 3320 cm^−1^, while other characteristic peaks remained almost unchanged (Fig. 1F), indicating the presence of hydrogen bonding interactions between BBR and LCDs [33,34]. UV-vis spectroscopy further revealed that LCDs exhibited a broad absorption band in the UV region, with a weak absorption peak at 280 nm attributable to the n→π* transition of oxygen-containing functional groups such as C=O on the carbon dot surface [35] (Fig. 1G). BBR displayed typical multiple absorption peaks at 270 nm, 345 nm, and 430 nm (Fig. 1G). The absorption spectrum of BLCDs was similar to that of BBR, indicating successful combination of BBR and LCDs, but with redshifted peaks and a hypochromic effect, showing absorption peaks at 290 nm, 365 nm, and 435 nm (Fig. 1G). The redshift and hypochromic effect of the UV absorption peaks suggested that BBR and LCDs coupled via hydrogen bonding [36], forming a new ground-state complex, which also led to significant fluorescence quenching of BLCDs (Fig. 1G–I). Visual inspection showed that the color of BLCDs powder and solution differed markedly from LCDs and BBR, and their fluorescence properties under UV light also varied, with BLCDs exhibiting significantly weaker fluorescence (Fig. 1H). Fluorescence spectroscopy data confirmed that the excitation and emission spectra of BLCDs differed from both LCDs and BBR, and the fluorescence intensity was quenched (Fig. 1I), further confirming that BLCDs are a new ground-state complex formed by the combination of BBR and LCDs. In addition, comprehensive ITC experiments characterized BLCDs formation under physiologically relevant conditions (Table S2). At pH 7.4 in PBS, BBR bound LCDs with K_D_ = 2.18 ± 0.19 μM and ΔG = −7.72 ± 0.16 kcal/mol, confirming spontaneous assembly. The entropy-driven binding (ΔH = 1.92 kcal/mol; TΔS = 9.64 kcal/mol) indicated electrostatic interactions with counterion release, while 150 mM NaCl shifted it to enthalpy-driven (ΔH = −3.18 kcal/mol; TΔS = 3.28 kcal/mol), revealing hydrogen bonding as a secondary force. This was confirmed by urea substantially reducing enthalpy (ΔH = −1.15 kcal/mol) and affinity (K_D_ = 85.40 μM). Thus, electrostatics drive BLCDs assembly with hydrogen bonding as a secondary contributor. Notably, BLCDs remained thermodynamically stable under acidic pH, high salt, and BSA (ΔG: −5.55 to −7.72 kcal/mol), with consistently negative ΔG values confirming considerable stability under physiological conditions. In summary, BBR and LCDs successfully assembled into BLCDs through non-covalent interactions such as electrostatic interactions and hydrogen bonding.

X-ray photoelectron spectroscopy (XPS) was used to analyze the primary compositional elements of LCDs and BLCDs. The results showed that the elemental contents of O, N, and C in LCDs were 38.11%, 1.62%, and 60.27%, respectively (Fig. 1J). Combined with FT-IR data (Fig. 1F), it could be inferred that the surface of LCDs was primarily composed of oxygen-containing functional groups, corresponding to the characteristic peak at 3423 cm^−1^ in the FT-IR spectrum attributed to O-H. Further peak fitting of the C 1s spectrum for LCDs revealed characteristic peaks at 284.1 eV, 286.2 eV, and 287.6 eV, corresponding to C-N/C-O (20.5%), C=C/C-C (62.3%), and C=O (17.2%) bonds, respectively. The N 1s spectrum showed a weak N-H (96.4%) characteristic peak at 399.5 eV, while the O 1s spectrum exhibited a characteristic peak for C-OH/C-O-C (99.5%) at 532.2 eV (Fig. 1J). These results indicated that the surface of LCDs was rich in oxygen-containing functional groups such as C=O and C-OH.

After binding with BBR, the elemental contents of O, N, and C in BLCDs were 20.42%, 4.53%, and 75.05%, respectively (Fig. 1K). Compared to LCDs, the N content in BLCDs significantly increased, while the O content decreased, indicating that the incorporation of BBR introduced nitrogen-containing groups. The C 1s spectrum of BLCDs showed characteristic peaks at 284.3 eV, 286.1 eV, and 287.2 eV, corresponding to C-N/C-O (56.1%), C-C (36.5%), and C=O (7.4%) bonds, respectively (Fig. 1K). The N 1s spectrum exhibited characteristic peaks at 395.8 eV (C=N, 5.6%), 400.8 eV (N-H, 81.8%), and 405.8 eV (Nitrogen oxides, 12.6%). The O 1s spectrum showed characteristic peaks at 531.3 eV (C-OH/C-O-C, 74.4%) and 532.8 eV (C=O, 25.6%). These results indicated that BBR binding altered the chemical composition of LCDs, significantly increasing the content of nitrogen-containing groups, further confirming the successful assembly of BBR and LCDs.

### 3.2 Investigation of the antioxidant and anti-inflammatory properties of BLCDs

**Fig. 2:**
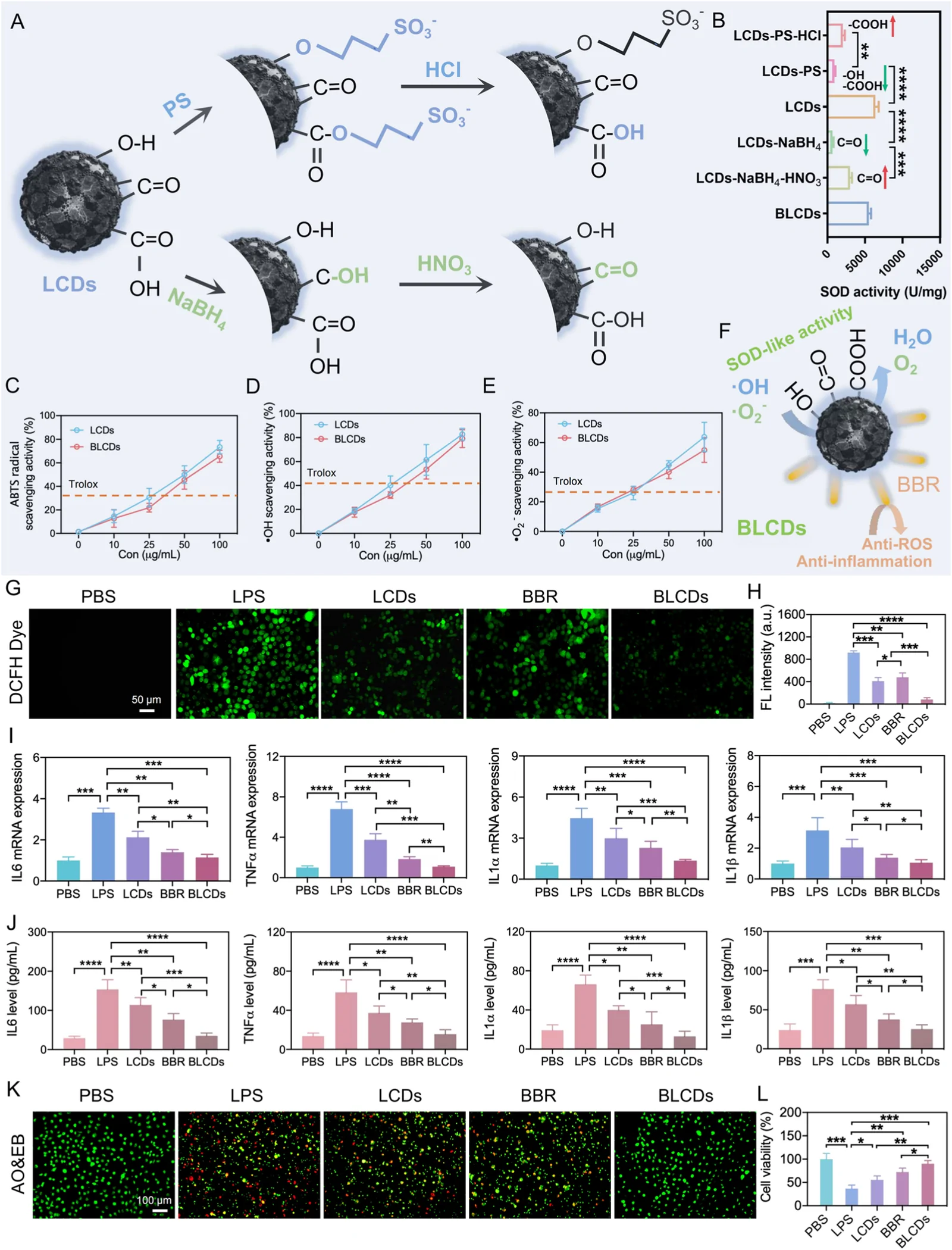
**Anti-ROS evaluationof BLCDs.** (A) Schematic diagram of LCDs modification. (B) Changes in SOD-like activity of LCDs before and after passivation, reduction, and re-oxidation treatments. (C) Total ROS scavenging capacity of LCDs and BLCDs assessed by the ABTS method (n = 3). (D) •OH scavenging activity determined by the salicylic acid method (n = 3). (E) •O_2_^−^ scavenging efficiency evaluated by the pyrogallol autoxidation method (n = 3). (F) Schematic illustration of the anti-ROS mechanism of BLCDs. (G) DCFH fluorescence staining images of LPS-stimulated NCM460 cells treated with LCDs (16.7 μg/mL), BBR (33.3 μg/mL), and BLCDs (50 μg/mL). The PBS group received only PBS. (H) Quantitative analysis of ROS levels (n = 3). (I) mRNA and (J) protein levels of inflammatory cytokines IL6, TNFα, IL1α, and IL1β (n = 3). (K) AO/EB staining images. (L) Statistical analysis of cell viability measured by the CCK-8 assay (n = 3).

Recent studies have shown that carbon dots possess superoxide dismutase (SOD)-like activity, in which oxygen-containing functional groups on their surface, such as carboxyl, hydroxyl, and carbonyl groups, play a key role [37,38]. These oxygen-containing functional groups can interact with ROS like •OH and •O_2_^−^, thereby promoting redox reactions. To investigate the role of surface oxygen-containing functional groups in the SOD-like activity of LCDs (Fig. 2A), we first measured the SOD-like activity of LCDs and BLCDs (Fig. 2B). The results showed that their SOD-like activities were 6.45 × 10^3^ U/mg and 6.03 × 10^3^ U/mg, respectively, with no significant difference, indicating that the binding of BBR to LCDs did not affect the SOD-like activity of LCDs (Fig. 2B).

Subsequently, we used 1,3-propanesultone (PS) to passivate carboxyl and hydroxyl groups [38] (Fig. 2A). PS reacts with carboxyl and hydroxyl groups on the carbon dot surface, forming ester and ether bonds, respectively, yielding the modified product LCDs-PS. Under acidic conditions, ester bonds can hydrolyze while ether bonds cannot. Therefore, after hydrolyzing LCDs-PS in 0.1 M HCl solution, LCDs with only hydroxyl groups passivated were obtained, named LCDs-PS-HCl (Fig. 2A). To characterize the surface functional groups of LCDs, LCDs-PS, and LCDs-PS-HCl, we performed FT-IR analysis. Compared with pristine LCDs, the FT-IR spectrum of LCDs-PS exhibited a markedly weakened absorption peak at 3430 cm^−1^, indicating a substantial reduction in hydroxyl groups after PS modification (Fig. S4A). In contrast, upon HCl treatment, the hydroxyl groups signal was largely restored. Meanwhile, the characteristic absorption bands of the sulfonic acid group (−SO_3_^−^) at 528, 610, and 1047 cm^−1^ appeared in the spectrum of LCDs-PS, and their intensities decreased significantly in that of LCDs-PS-HCl (Fig. S4A). These combined observations confirm the successful modification of LCDs with PS.

Activity determination results showed that the SOD-like activity of LCDs-PS decreased to 1.15 × 10^3^ U/mg, indicating that the passivation of hydroxyl and carboxyl groups significantly reduced the SOD-like activity of LCDs (Fig. 2B). For LCDs-PS-HCl, with carboxyl groups restored while hydroxyl groups remained passivated, the SOD-like activity recovered to 2.53 × 10^3^ U/mg but failed to return to the original level (Fig. 2B). This suggested that hydroxyl groups also play a key role in the SOD-like activity of LCDs.

To further explore the role of carbonyl groups in the SOD-like activity of LCDs, we used NaBH_4_ to reduce the carbonyl groups on the LCDs surface (Fig. 2A). XPS analysis of LCDs-NaBH_4_ and LCDs-NaBH_4_-HNO_3_ revealed that the C=O content dropped from 17.2% to 5.4% after NaBH_4_ reduction, and rebounded to 15.0% after HNO_3_ treatment, confirming the effective regulation of surface carbonyl groups through sequential reduction and oxidation (Fig. S4B and C). The SOD-like activity of the resulting LCDs-NaBH_4_ decreased significantly to 0.69 × 10^3^ U/mg, indicating that carbonyl groups play an important role in the SOD-like activity of LCDs (Fig. 2B). To verify this conclusion, we subsequently treated with HNO_3_ to promote carbonyl regeneration. The results showed that the SOD-like activity of LCDs-NaBH_4_-HNO_3_ significantly recovered to 2.84 × 10^3^ U/mg, further confirming the contribution of carbonyl groups to the SOD-like activity of LCDs (Fig. 2B). In summary, these results indicated that oxygen-containing functional groups such as carboxyl, hydroxyl, and carbonyl groups are key factors for LCDs to exert SOD-like activity.

We evaluated the total ROS scavenging efficiency of LCDs and BLCDs at various concentrations *in vitro*. Both LCDs and BLCDs exhibited significant, concentration-dependent ROS scavenging activity, with no notable difference between them (Fig. 2C), consistent with the SOD-like activity results (Fig. 2B). This indicated that BBR conjugation did not compromise the ROS scavenging capacity of LCDs. To further investigate the SOD-like activity of LCDs and BLCDs, we examined their *in vitro* scavenging efficiency against •OH and •O_2_^−^ at different concentrations (Fig. 2D and E). Similarly, both LCDs and BLCDs scavenged •OH and •O_2_^−^ significantly in a concentration-dependent manner, without significant intergroup differences, further confirming their SOD-like activity (Fig. 2D and E). In summary, BLCDs inherited the SOD-like activity of LCDs and could exert significant ROS scavenging ability against •OH and •O_2_^−^ through oxygen-containing functional groups such as carboxyl, hydroxyl, and carbonyl on their surface.

BLCDs not only possessed the nanoscale characteristics of LCDs but also carried BBR with significant anti-inflammatory activity (Fig. 2F). As a multi-target drug, BBR can inhibit the expression of pro-inflammatory cytokines such as TNFα, IL6, IL1α, and IL1β by regulating multiple signaling pathways including NF-κB, NRF2, EIF2AK2, JAK/STAT, AKT, and NLRP3 [[13], [14], [15], [16], [17], [18]], thereby effectively reducing oxidative stress levels *in vivo*. Given that Zanglian Wan is widely used clinically for the treatment of inflammatory bowel disease, we first established an *in vitro* enteritis cell model using LPS-induced NCM460 cells and treated them with LCDs, BBR, and BLCDs. The results showed that, unlike the ROS scavenging results *in vitro*, the antioxidant capacity of BLCDs within cells was significantly superior to that of LCDs (Fig. 2G and H). This was attributed to the indirect antioxidant effect of BBR within cells through its anti-inflammatory mechanisms, while BLCDs possessed both the SOD-like activity of LCDs and the natural anti-inflammatory activity of BBR, with the two acting synergistically to exhibit more significant ROS scavenging ability in inflammatory cells (Fig. 2F–H).

In terms of intracellular antioxidation, the direct ROS scavenging effect of LCDs was more efficient than the indirect inhibitory effect of BBR. Regarding anti-inflammation, we found that BBR had a more significant ability to inhibit the expression of pro-inflammatory factors, substantially downregulating the mRNA and protein levels of IL6, TNFα, IL1α, and IL1β, which was closely related to its multi-target regulatory natural anti-inflammatory pharmacological effects (Fig. 2I and J). Notably, BLCDs also inherited the natural anti-inflammatory efficacy of BBR while simultaneously achieving synergistic regulation of inflammation through the antioxidant mechanism of LCDs. Compared with LCDs or BBR alone, BLCDs exhibited a more significant regulatory effect on pro-inflammatory factors, restoring the inflammation level in LPS-induced NCM460 cells to a level close to that of the PBS group (Fig. 2I and J). Owing to the synergistic anti-inflammatory effect of BBR and the antioxidant activity of LCDs, BLCDs significantly restored the viability of LPS-stimulated NCM460 cells (Fig. 2K). Moreover, treatment with various concentrations of LCDs or BLCDs (0-100 μg/mL) exhibited no obvious cytotoxicity, indicating good biosafety (Fig. S5A and B). EC_50_ determination of BBR, LCDs, and BLCDs in these cells further demonstrated that the differences in their biological effects were not due to high-concentration non-specific effects (Fig. S6). We then assessed BLCDs stability and BBR release by measuring dialysate BBR content under simulated gastrointestinal pH conditions (1.2, 6.8, and 7.4). We found that BLCDs showed good stability within 6 h at pH 1.2, and remained stable at pH 6.8 and 7.4 over 72 h (Fig. S7A). We also observed that BLCDs improved cellular BBR uptake by approximately 4-fold at 72 h compared with free BBR (Fig. S7B). We noted that the BBR + LCDs mixture may also form complexes during prolonged incubation that moderately enhance uptake; however, we confirmed that the efficiency remained markedly lower than that of BLCDs, highlighting the superiority of the integrated formulation.

To further compare the synergistic effects, we treated LPS-stimulated NCM460 cells with multiple dose gradients of BBR, LCDs, and BLCDs (1.5625, 3.125, 6.25, 12.5, 25, 50, and 100 μg/mL), and constructed full dose-effect curves (Fig. S8). The combination index (CI) was calculated using CompuSyn software to classify the interactions as synergistic (CI < 1), additive (CI = 1), or antagonistic (CI > 1) [39]. At a fraction affected (Fa) of 0.5, the CI values were determined to be 0.24 for BLCDs and 0.43 for the BBR + LCDs combination, indicating that both formulations exert synergistic effects, and notably, the synergy of BLCDs is significantly superior to that of the equimolar physical mixture of BBR and LCDs.

In summary, BLCDs possessed both the SOD-like activity of LCDs and the natural anti-inflammatory capacity of BBR, representing a nanomedicine with significant research value inspired by Zanglian Wan.

### 3.3 RNA-seq revealed the mechanism of BLCDs in treating UC

BLCDs possessed the dual functions of ROS scavenging and inhibition of inflammatory factors, which were actually synergistic and mutually regulating within cells. ROS scavenging could inhibit the activation of inflammatory factor pathways, while the inhibition of inflammatory factors helped reduce intracellular oxidative stress levels. Therefore, we aimed to delve into the anti-inflammatory and antioxidant mechanisms of BLCDs in LPS-induced NCM460 cells using transcriptomics, identify key regulatory molecules through which BLCDs exerted their pharmacological effects, and pinpoint the core targets of their anti-inflammatory and antioxidant actions.

**Fig. 3:**
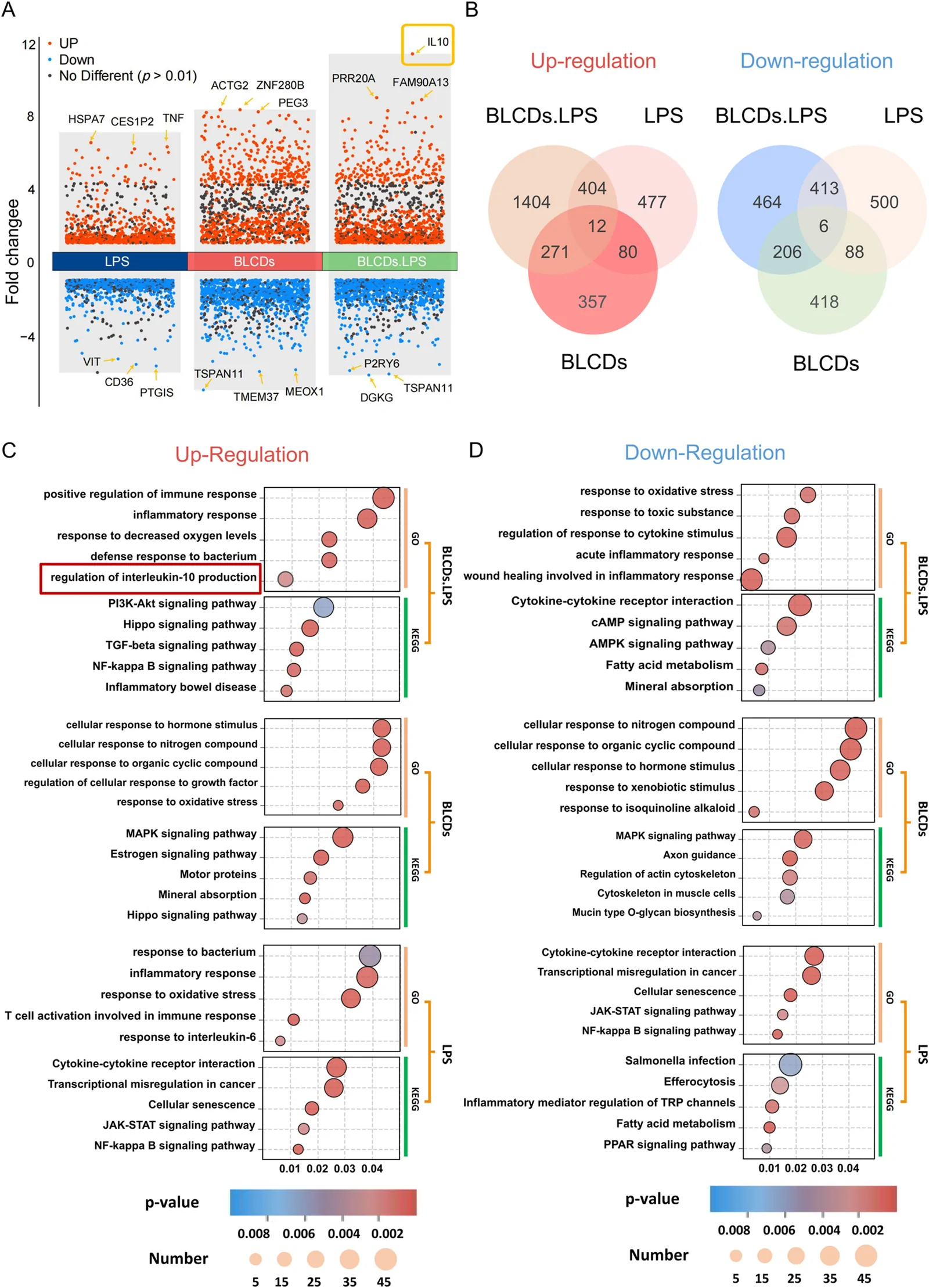
**RNA-seq analysis of LPS-induced NCM460 cells before and after BLCDs treatment.** (A) Volcano plots of DEGs. LPS: LPS-induced NCM460 cells; BLCDs: BLCDs-treated NCM460 cells; BLCDs. LPS: LPS-induced NCM460 cells treated with BLCDs. (B) Venn diagram analysis. (C) GO and KEGG enrichment analysis of up-regulated DEGs. (D) GO and KEGG enrichment analysis of down-regulated DEGs.

Through RNA-seq mechanistic analysis of LPS-induced NCM460 cells before and after BLCDs treatment, we found that IL10 was the most significantly upregulated gene (approximately 11.5-fold) after BLCDs treatment (Fig. 3A and B), and the regulation of IL10 production pathway was significantly enriched (Fig. 3C). Furthermore, among the upregulated genes in BLCDs-treated LPS-induced NCM460 cells (BLCDs.LPS group), inflammatory response and response to decreased oxygen levels were significantly enriched, suggesting that BLCDs treatment activated intracellular anti-inflammatory responses and reduced oxidative stress levels (Fig. 3C). Among the downregulated genes, response to oxidative stress, response to toxic substance, and regulation of response to cytokine stimulus were highly enriched, further confirming the antioxidant and anti-inflammatory effects of BLCDs (Fig. 3D). Additionally, we separately examined the impact of BLCDs on the transcriptome of NCM460 cells. The results showed that after exposure to BLCDs, cells primarily exhibited enrichment in pathways related to hormone regulation, response to nitrogen compound, and response to cyclic compound, indicating that normal cells mainly underwent absorption and metabolism of BLCDs (Fig. 3C and D), which was consistent with the results of cell viability assays (Fig. S5B).

Given that IL10 was the most significantly upregulated gene in the BLCDs.LPS group and regulation of IL10 production was highly enriched (Fig. 3C), we conducted an in-depth analysis of this pathway. Transcriptomic radar charts showed that while IL10 increased slightly upon LPS induction, its expression was substantially upregulated following BLCDs intervention (Fig. S9A). We further verified the RNA-seq results by measuring IL10 mRNA and protein expression levels (Fig. S9B). Furthermore, within this pathway, the IL10 downstream target molecule NLRP3 was significantly upregulated (7.6-fold) after LPS induction, whereas its expression significantly decreased after BLCDs treatment (Fig. S9A). Detection of NLRP3 mRNA and protein levels confirmed that BLCDs could substantially reverse the LPS-induced increase in NLRP3 (Fig. S9C). Simultaneously, we also measured the expression of downstream effector pro-inflammatory cytokines IL1β and IL18 regulated by NLRP3 (Fig. S9D). The results showed that BLCDs intervention significantly decreased the LPS-induced expression of IL1β and IL18, further supporting that BLCDs regulated NLRP3 expression through the IL10 pathway. The increase in IL10 and decrease in NLRP3 indicated a reduction in cellular inflammation levels. We measured the expression of classic inflammatory cytokines TNFα and IL6 (Fig. S9E), confirming the significant alleviating effect of BLCDs on inflammation.

To delineate the individual contributions of each component to IL10 induction, we evaluated IL10 expression in LPS-stimulated NCM460 cells treated with LCDs, BBR, the physical mixture of BBR and LCDs, or BLCDs (Fig. S10). Among all treatments, BLCDs induced the most potent upregulation of IL10 expression. Both BBR and LCDs alone exhibited moderate inductive effects, with BBR showing significantly stronger activity than LCDs and playing a dominant role in the combination.

Concurrently, we systematically examined the expression and activation status of upstream transcription factors, including NF-ĸB (p65), STAT3, AP-1 (c-Jun), and C/EBP-β. The results revealed that BLCDs treatment markedly upregulated the phosphorylation level of STAT3 (p-STAT3), suggesting that STAT3 activation played a pivotal role in the transcriptional regulation of IL10 (Fig. S11A). Based on these findings, we further investigated the impact of Stattic, a specific inhibitor of p-STAT3, on the IL10/NLRP3 pathway. Inhibition of p-STAT3 significantly downregulated IL10 expression while restoring NLRP3 expression (Fig. S11B), strongly confirming that BLCDs-induced IL10 upregulation is mediated through p-STAT3 activation.

To further demonstrate that BLCDs exerted their anti-inflammatory and antioxidant functions through IL10 *in vitro*, we performed IL10 inhibition experiments on RAW264.7 cells, which were more sensitive to inflammation regulation (Fig. S12). Co-treatment of LPS-induced RAW264.7 cells with BLCDs and JES5-2A5 (an IL10 inhibitor) showed that the expression level of the anti-inflammatory cytokine IL10 was significantly inhibited (Fig. S12A), while downstream NLRP3 and its effector pro-inflammatory cytokines IL1β and IL18 were significantly upregulated (Fig. S12B and C). Consequently, the cellular inflammation level was not alleviated, with TNFα and IL6 remaining highly expressed (Fig. S12D). This result indicated that BLCDs exerted their key anti-inflammatory effects by regulating NLRP3 and its downstream pro-inflammatory cytokines through IL10. We further investigated the impact of graded concentrations of BLCDs on IL10 expression in LPS-stimulated and unstimulated NCM460 and RAW264.7 cells (Fig. S13). IL10 levels were found to rise with increasing BLCD concentrations, suggesting that BLCDs efficiently induce IL10 production and exhibit potent anti-inflammatory activity. Additionally, detection of cellular ROS fluorescence intensity also demonstrated that BLCDs exerted antioxidant effects via IL10 (Fig. S12E), and inhibiting IL10 significantly weakened the ability of BLCDs to rescue LPS-induced cells (Fig. S12F). MDA and GSH levels were measured to evaluate the anti-oxidative stress effect of BLCDs on LPS-induced cells (Fig. S14). The results showed that LPS stimulation significantly increased MDA levels and decreased GSH content, indicating a marked oxidative stress response (Fig. S14 A and B). Following treatment with various agents, the cellular oxidative stress status was substantially reversed, with the BLCDs group exhibiting the most pronounced improvement. These findings demonstrated that the combination of BBR and LCDs exerted a synergistic effect, which was superior to either compound alone. Notably, BLCDs also outperformed the equimolar physical mixture of BBR and LCDs, further supporting the advantage of the formulated combination.

In summary, BLCDs, possessing both anti-inflammatory and antioxidant functions, primarily exerted their effects in inflammatory cells by regulating the IL10 pathway.

### 3.4 Evaluation of the therapeutic efficacy of BLCDs in UC

**Fig. 4:**
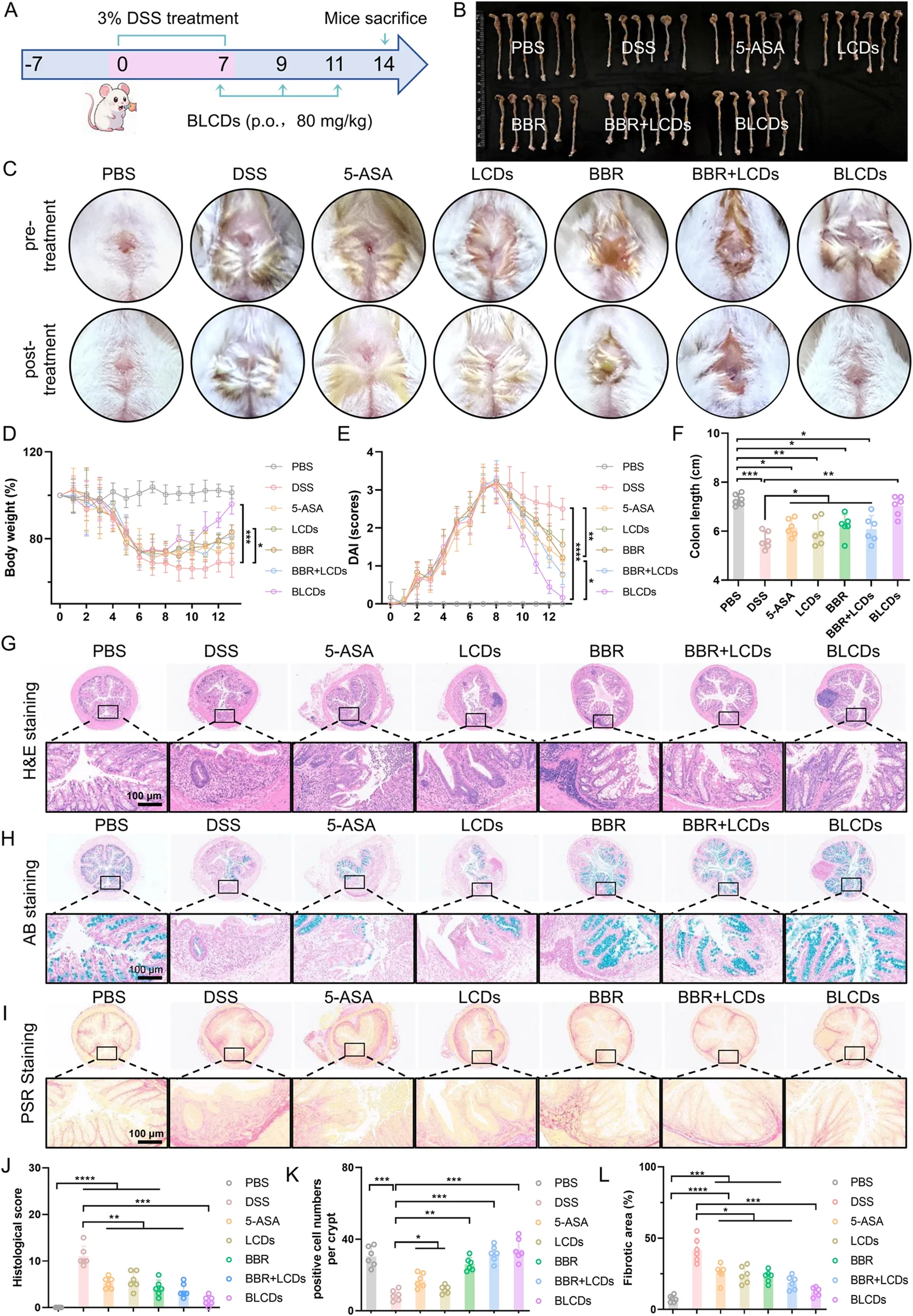
**Evaluation of the therapeutic effect of BLCDs on DSS-induced mice.** (A) Schematic diagram of the animal treatment protocol with BLCDs. (B) Visual representation of dissected colons. (C) Anal conditions of mice before and after treatment. (D) Body weight changes in mice (n = 6). (E) DAI scores (n = 6). (F) Statistical analysis of colon length (n = 6). (G) H&E staining, (H) AB staining, and (I) PSR staining of mouse colons from each group. Black boxes indicate magnified regions. (J) Pathological scoring results (n = 6). (K) Quantitative analysis of goblet cells in colonic crypts of each group (n = 6). (L) Quantitative results of colonic fibrosis area in mice (n = 6).

To assess the *in vivo* therapeutic effect of BLCDs on inflammatory bowel disease, we established a mouse model of UC using 3% DSS administered via free drinking water. UC mice were treated with 5-ASA (positive control), LCDs, BBR, a mixture of BBR and LCDs (BBR + LCDs), and BLCDs (Fig. 4A). Prior to formal treatment, a dose-response experiment determined the optimal dosage of BLCDs, showing that 80 mg/kg BLCDs yielded the best DAI score; thus, this dose was selected for subsequent experiments (Fig. S15). PBS-treated healthy mice served as control, and DSS-induced UC mice served as the model group. Mice were euthanized on day 14, and major organs were dissected and collected for subsequent analysis.

Dissection images of mouse colons showed that the DSS model group had the shortest colon length, while the BLCDs treatment group exhibited the longest colon length, close to that of the PBS healthy control group (Fig. 4B). Anal photographs taken before and after treatment revealed significant wet tail (perianal dampness due to diarrhea) in the DSS group (Fig. 4C). Then, we evaluated the perianal images using objective quantitative criteria. The DSS group had the highest contamination score, while the BLCDs group had the lowest, close to the healthy group level (Fig. S16). All treatment groups showed varying degrees of improvement, with the BLCDs group demonstrating the most significant therapeutic effect, essentially returning to normal levels (Fig. 4B and C). The BBR + LCDs group ranked second in therapeutic efficacy, indicating that simple co-administration was less effective against UC than the integrated BLCDs nanomedicine (Fig. 4B and C). This superiority underscores the advantage of BLCDs as a holistic nano-formulation for more precise and efficient anti-inflammatory action, a finding further supported by measurements of body weight (Fig. 4D), DAI scores (Fig. 4E), and colon length (Fig. 4F). Notably, BLCDs were superior to the positive drug 5-ASA and its individual components (LCDs and BBR alone) in restoring mouse body weight (Fig. 4D), improving DAI scores (Fig. 4E), and restoring colon length (Fig. 4F). These data not only confirmed the *in vivo* therapeutic safety of BLCDs but also highlighted the synergistic advantage of BLCDs, combining the antioxidant properties of LCDs with the natural anti-inflammatory activity of BBR, in treating UC.

To further explore the therapeutic effect of BLCDs on UC, we performed pathological evaluation (Fig. 4H), goblet cell counting (cells that secrete mucin to maintain the intestinal mucosal barrier) (Fig. 4G), and intestinal fibrosis analysis on colon tissues from each group of mice (Fig. 4I). The results showed that mice in the BLCDs treatment group had colon pathology scores (Fig. 4G and J), positive goblet cell counts (Fig. 4H and K), and fibrotic areas closest to those of the PBS healthy control group (Fig. 4I and L), demonstrating the most significant efficacy among all treatment groups.

**Fig. 5:**
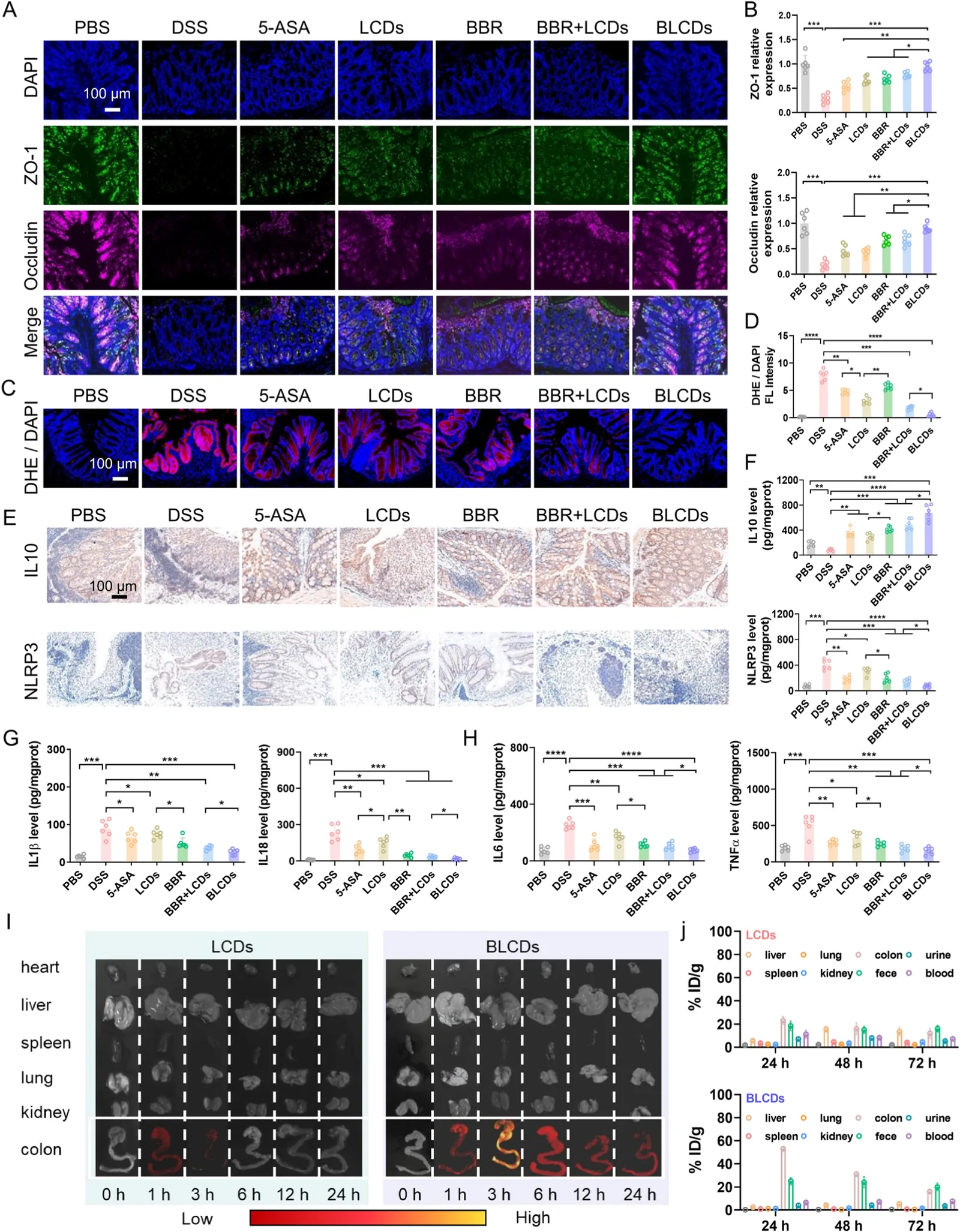
**Mechanistic analysis of BLCDs treatment.** (A) Immunofluorescence staining of DAPI, ZO-1, and Occludin in colon tissues from each group. (B) Quantitative analysis of ZO-1 and Occludin expression levels (n = 6). (C) DHE/DAPI staining of colon tissues. (D) Quantitative analysis of the DHE/DAPI fluorescence intensity ratio (n = 6). (E) Immunohistochemical staining results for IL10 and NLRP3. (F) Expression levels of IL10 and NLRP3 (n = 6). (G) Expression levels of IL1β and IL18 (n = 6). (H) Expression levels of IL6 and TNFα (n = 6). (I) Near-infrared fluorescence imaging of major mouse tissues at different time points (0, 1, 3, 6, 12, 24 h) after administration of Cy5.5-labeled LCDs and BLCDs. (J) Biodistribution of LCDs and BLCDs at 24, 48, and 72 h (n = 6).

Furthermore, we examined the expression levels of key molecular markers reflecting intestinal tight junction integrity, permeability, and barrier function, namely ZO-1 and Occludin (Fig. 5A). The results indicated that BLCDs could significantly reverse DSS-induced intestinal barrier dysfunction, substantially upregulating the expression of ZO-1 and Occludin, with efficacy superior to other treatment groups (Fig. 5A and B; Fig. S17).

Detection of oxidative stress levels in intestinal tissue showed that the DHE fluorescence intensity was significantly reduced in the BLCDs group, indicating that BLCDs could effectively inhibit intestinal oxidative stress levels in UC mice (Fig. 5D). These results were consistent with those from *in vitro* cellular experiments. They suggest that BLCDs not only inherit the SOD-like activity of LCDs but also, through the loaded BBR, inhibit ROS generation via anti-inflammatory pathways. Consequently, BLCDs demonstrate a more potent ROS scavenging capability *in vivo* than the individual components. Additionally, H&E staining results showed that BLCDs caused no significant toxic damage to major organs (heart, liver, spleen, lungs, kidneys) of mice during treatment (Fig. S18).

To confirm the *in vivo* relevance of our RNA-seq and *in vitro* findings, we examined the expression of IL10, its downstream target NLRP3, and the effector cytokines IL1β and IL18 in colon tissues from each group using ELISA assays. The results showed that BLCDs could significantly upregulate IL10 expression in DSS-induced mice (Fig. 5E and F), thereby inhibiting the expression of its downstream target gene NLRP3 and the effector pro-inflammatory cytokines IL1β and IL18 (Fig. 5E–G). We further assessed *caspase-1* expression in the colon tissues and found that its levels were positively correlated with those of IL1β and IL18 across all groups. Notably, BLCDs most effectively suppressed the DSS-induced upregulation of *caspase-1* (Fig. S19A), further supporting the inhibitory effect of BLCDs on the NLRP3 inflammasome pathway. This favorable shift in the inflammatory microenvironment drove inflammation levels in DSS-induced mice toward those of the PBS group, effectively restoring normal physiological status (Fig. 5H). Furthermore, Western Blot analysis of the STAT3-IL10-NLRP3 pathway revealed that DSS markedly upregulated NLRP3, whereas BLCDs most effectively reversed this effect (Fig. S17). This reversal was attributed to a pronounced increase in p-STAT3-mediated IL10 expression, which subsequently suppressed NLRP3 to levels comparable to those in the PBS group (Fig. S17). BLCDs exhibited superior modulatory effects on this pathway over all other treatments, highlighting the advantage of the integrated administration of BBR and LCDs as a single formulation.

To investigate the *in vivo* distribution and metabolism of LCDs and BLCDs, we labeled LCDs and BLCDs with Cy5.5 fluorescent dye using the EDC/NHS method. Near-infrared fluorescence imaging results showed that after oral administration, LCDs and BLCDs were primarily enriched in intestinal tissue (Fig. 5I). Notably, BLCDs reached their peak enrichment in the intestine 3 h post-administration, with residual accumulation still present at 24 h (Fig. 5I). In contrast, LCDs peaked at 1 h, and their metabolic clearance rate from the intestine was significantly faster than that of BLCDs (Fig. 5I). Within the 24-72 h time window, the distribution of BLCDs and LCDs in the intestine gradually decreased, with partial excretion via feces and urine (Fig. 5J). We also performed a pharmacokinetic study in healthy mice given a single oral dose of BLCDs. We collected colon tissues at multiple time points and quantified BBR levels in each sample. We found that the elimination half-life (t_1/2_) of BLCDs in colon tissues was 36.9 h, supporting our every-other-day dosing regimen (Fig. S20G).

In summary, BLCDs exhibited prolonged intestinal retention, fully leveraging the antioxidant properties of LCDs and the anti-inflammatory activity of BBR. They significantly upregulated IL10 expression and inhibited NLRP3 production along with the secretion of pro-inflammatory cytokines. This effectively alleviated intestinal oxidative stress in DSS-induced mice and preserved intestinal barrier integrity and function, thereby demonstrating a significant therapeutic effect on UC.

### 3.5 Effects of BLCDs on gut microbiota and metabolites in UC

**Fig. 6:**
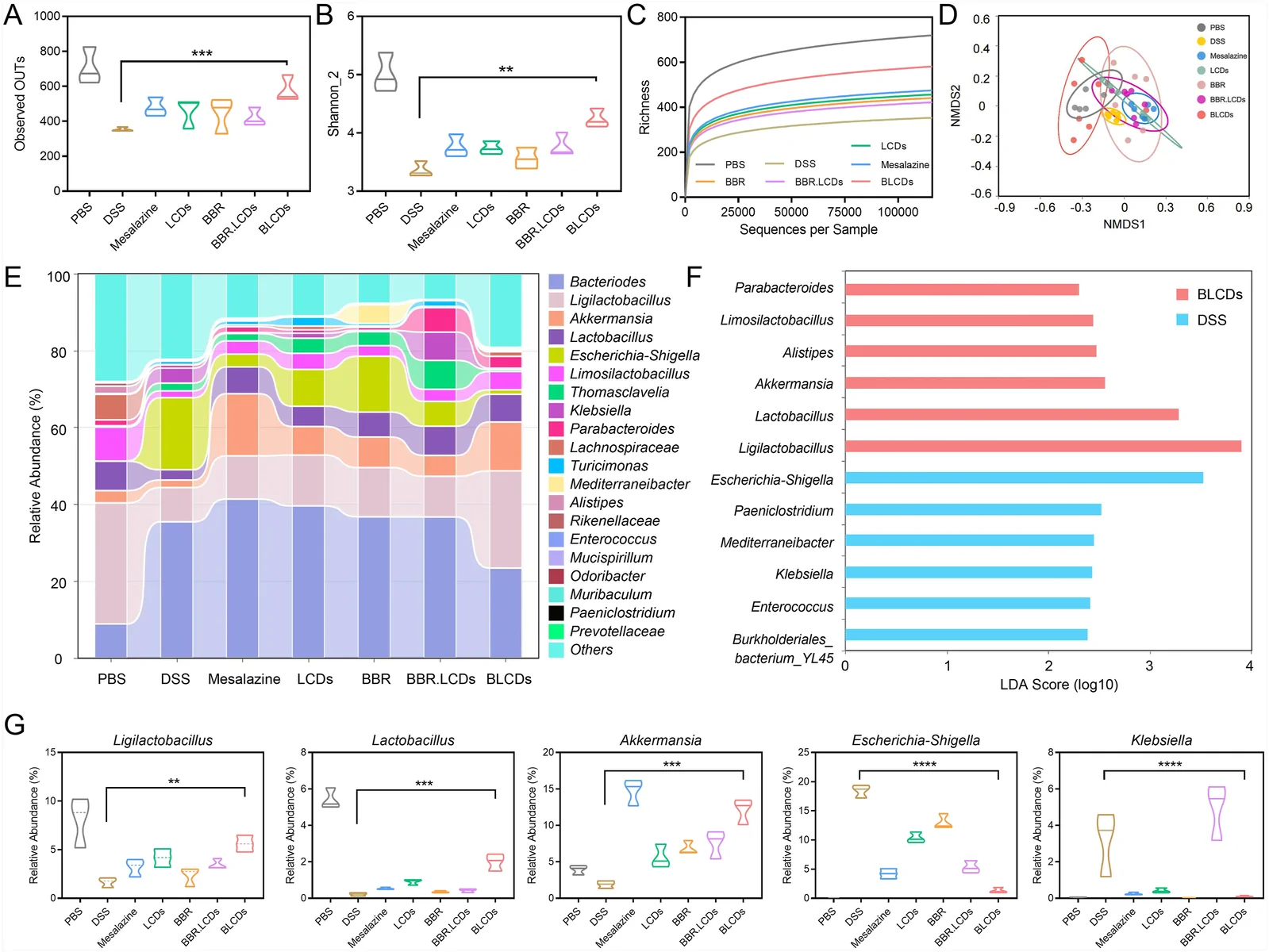
**Effects of BLCDs on the gut microbiota of DSS-induced mice.** Evaluation of gut microbial communities by (A) observed OTUs, (B) Shannon index, (C) Richness index, and (D) NMDS2 analysis. (E) Genus-level relative abundance as a percentage of total sequences. (F) LDA scores for the BLCDs group versus the DSS group. (G) Relative abundance of *Ligilactobacillus*, *Lactobacillus*, *Akkermansia*, *Escherichia-Shigella*, and *Klebsiella* (n = 6).

Changes in the gut microbiota were important indicators for evaluating the therapeutic effect of UC. To delve into the impact of BLCDs on the gut microbiota, we performed 16S rDNA sequencing on fecal samples from mice in each group. Analysis of the sequencing data showed that BLCDs intervention significantly increased the abundance (observed OTUs) (Fig. 6A), diversity (Shannon curve) (Fig. 6B), and richness (rarefaction curve) of the gut microbiota (Fig. 6C). Nonmetric multidimensional scaling (NMDS) based on microbial community composition revealed that the gut microbiota profile of the BLCDs group was significantly different from that of the DSS model group and other treatment groups (Fig. 6D).

From the relative abundance of gut microbiota and LDA scores, it was evident that after BLCDs treatment, beneficial bacteria such as *Ligilactobacillus*, *Lactobacillus*, and *Akkermansia* significantly increased, while harmful bacteria such as *Escherichia-Shigella* and *Klebsiella* substantially decreased (Fig. 6E and F). Notably, the positive regulatory effect of BLCDs on the gut microbiota was significantly superior to that of the positive drug 5-ASA (mesalazine) (Fig. 6G).

**Fig. 7:**
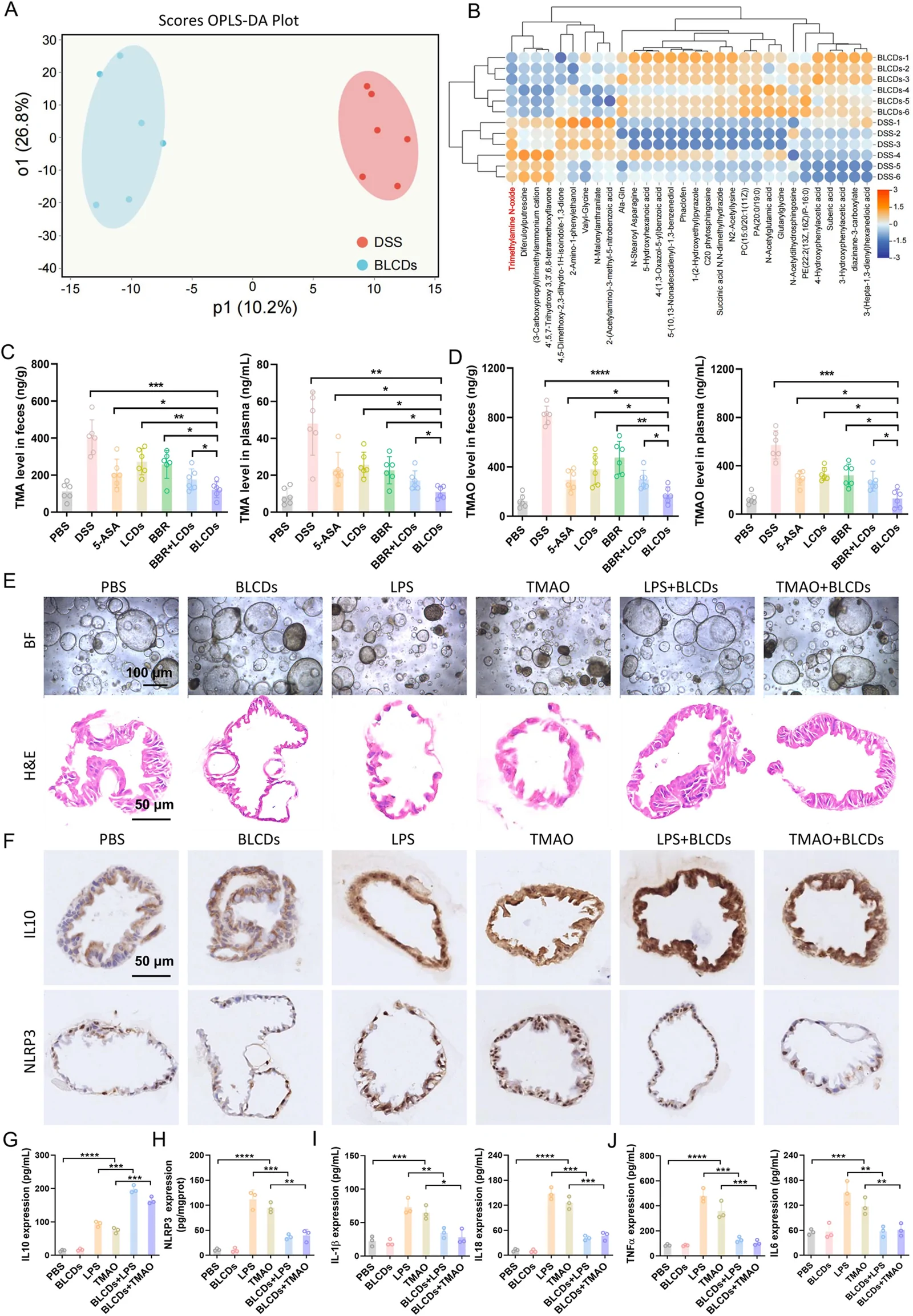
**Effects of BLCDs on intestinal metabolites in DSS-induced mice.** (A) OPLS-DA score plot between the DSS and BLCDs groups (n = 6). (B) Heat map of differential metabolite aggregation in mice from the DSS and BLCDs groups (n = 6). (C) TMA levels in feces and plasma (n = 6). (D) TMAO levels in feces and plasma (n = 6). (E) Bright-field images and H&E staining of CNOs treated with PBS, BLCDs, LPS, TMAO, LPS + BLCDs, and TMAO + BLCDs. (F) Immunohistochemical staining for IL10 and NLRP3 in CNOs from each group. (G) Expression levels of IL10 and (H) NLRP3 (n = 6). (I) Expression levels of IL1β and IL18. (J) Expression levels of TNFα and IL6 (n = 6).

Changes in the gut microbiota could further influence the composition of intestinal metabolites, thereby regulating UC disease progression. We examined intestinal metabolites in mice from all groups and found significant differences in metabolite profiles (Fig. 7A, Fig. S21A). A heatmap of intestinal metabolites showed that after BLCDs treatment, the levels of trimethylamine N-oxide (TMAO) in the intestines of all mice were significantly reduced, whereas they were significantly elevated in the DSS group (Fig. 7B, Fig. S21B). TMAO was a key molecule metabolized by gut microbes, primarily generated by the oxidation of trimethylamine (TMA), and could be distributed throughout the body via the bloodstream [40]. Elevated TMAO levels could induce intestinal inflammation, oxidative stress, and DNA damage, promoting the progression of intestinal lesions towards a precancerous state. Research indicated that *Klebsiella* was involved in TMA metabolism [41,42], consistent with our observation of reduced *Klebsiella* abundance in the BLCDs group (Fig. 6F and G). Therefore, we further measured TMA and TMAO levels in feces and plasma. The results showed that both TMA and TMAO in feces and plasma were significantly downregulated after BLCDs treatment (Fig. 7C and D). TMAO is mainly produced in the liver by *FMO3*-mediated oxidation of TMA and then released into the plasma to affect peripheral organs including the intestine. Hepatic *FMO3* mRNA levels were then assessed across groups. DSS challenge markedly elevated *FMO3* expression, while BLCDs treatment caused a substantial reduction, with BLCDs showing significantly greater inhibition than all other groups (Fig. S19B). This trend closely paralleled the TMA and TMAO levels in feces and plasma, further supporting the role of BLCDs in regulating TMAO metabolism. These results suggested that BLCDs intervention could alter the expression of the intestinal metabolite TMAO in DSS-induced mice by modulating the gut microbiota, thereby enhancing the therapeutic effect on UC. We further quantified the colonic expression of TMA-generating enzymes (CutC/D) in each group (Fig. S22A and B), confirming that BLCDs inhibited the gut microbial-TMA synthesis pathway, which mechanistically supports the observed downregulation of TMAO. To further substantiate the *in vivo* regulation of the *Klebsiella*-TMAO axis by BLCDs, we employed germ-free mice colonized with *Klebsiella* (ATCC 43816) (Fig. S22C). Specifically, BLCDs and 3,3-dimethyl-1-butanol (DMB), a known CutC/D inhibitor, were administered as a positive control to the model mice. The results demonstrated that BLCDs, similar to DMB, significantly suppressed the *Klebsiella* burden and markedly downregulated the expression levels of CutC/D, TMA, and TMAO, indicating that BLCDs reduced TMAO production through interference with the TMA generation pathway (Fig. S22D–H). Collectively, the *Klebsiella* colonization experiment, together with the measured levels of CutC/D, TMA, and TMAO, strongly demonstrated that BLCDs exerted their therapeutic effects against UC via modulation of the *Klebsiella*-TMAO axis.

To validate the regulatory effect of BLCDs on TMAO, we conducted experiments using colorectal normal organoids (CNOs). The results showed that TMAO treatment, similar to LPS treatment, significantly reduced the diameter and viability of CNOs while inducing a pronounced inflammatory phenotype (Fig. 7E, Fig. S23A and B). In contrast, BLCDs treatment effectively reversed these effects in both LPS- and TMAO-induced organoids, restoring diameter and viability (Fig. S23A and B), reducing oxidative stress levels (Fig. S23C), and improved histological scores along with alleviated inflammatory lesions (Fig. 7E, Fig. S24).

Mechanistically, TMAO stimulation significantly upregulated the expression of NLRP3 and its downstream pro-inflammatory cytokines IL1β and IL18, identifying TMAO as a key metabolite in exacerbating intestinal inflammation (Fig. 7F–H and J). More importantly, BLCDs treatment significantly induced IL10 production in LPS/TMAO-induced CNOs, which in turn downregulated NLRP3 and its effector cytokines (Fig. 7F–I). This ultimately ameliorated the inflammatory microenvironment, as evidenced by decreased expression of TNFα and IL6 (Fig. 7J).

To fully verify that TMAO-mediated intestinal inflammation could indeed be blocked by IL10 regulated by BLCDs, we performed IL10 inhibition experiments (Fig. S25). Co-incubating BLCDs with AS101 (an IL10 inhibitor) in TMAO-induced CNOs resulted in a significant reversal of BLCDs efficacy. Under AS101 intervention, the promoting effect of BLCDs on IL10 production was inhibited (Fig. S25A and B), leading to failure in restoring the diameter and viability of TMAO-induced CNOs (Fig. S25C–E), which maintained high oxidative stress levels (Fig. S25F).

In summary, BLCDs could regulate the abundance of gut microbiota in UC mice, significantly reducing the production of the intestinal inflammation-related metabolite TMAO. Simultaneously, BLCDs could inhibit TMAO-mediated inflammatory responses through the regulation of IL10, thereby effectively ameliorating UC disease progression.

### 3.6 *In vivo* safety evaluation of BLCDs

Finally, we systematically evaluated the *in vivo* safety of BLCDs (Fig. S20A). Healthy mice treated with PBS served as controls. On day 7, major organs and blood samples were collected from the mice for analysis. Concurrently, we designed a long-term safety observation experiment, which showed no significant difference in body weight changes over 60 days between the BLCDs group and the PBS group (Fig. S20B). H&E staining results indicated that BLCDs treatment caused no obvious toxic damage to the heart, liver, spleen, lungs, kidneys, or intestines of mice (Fig. S20C). Complete blood count analysis revealed that BLCDs administration did not induce abnormal changes in the counts of white WBC, PLT, HGB, or RBC (Fig. S20D). Serum biochemical markers further demonstrated that BLCDs had no significant impact on mouse liver function (ALP, ALT, AST) or kidney function (BUN, Cr, UA) (Fig. S20E). Additionally, *in vitro* hemolysis assays confirmed that BLCDs exhibited no hemolytic toxicity towards mouse red blood cells (Fig. S20F). Taken together, BLCDs possessed good *in vivo* safety.

## 4 Discussion

This study, inspired by the traditional formula Zanglian Wan, successfully constructed a nano-assemblies BLCDs formed by the self-assembly of LCDs and BBR. It systematically elucidated the mechanism by which BLCDs ameliorate UC through the regulation of IL10 and TMAO.

Zanglian Wan, a classic formula clinically used for inflammatory bowel disease, involves the co-processing of *Coptis chinensis Franch.* and carbonized *Sanguisorba officinalis L.* Inspired by this, we performed *in vitro* self-assembly of the active nano-component LCDs derived from carbonized *Sanguisorba officinalis L.* with BBR, the main component of *Coptis chinensis Franch.* Characterization results showed that LCDs had an average particle size of 8.5 nm and a surface rich in oxygen-containing functional groups such as carboxyl, hydroxyl, and carbonyl groups (Fig. 1B–F and J). When mixed with BBR at a 2:1 ratio, they successfully self-assembled through electrostatic interactions and hydrogen bonding to form BLCDs with a particle size of approximately 72.4 nm (Fig. 1C–I). TEM, FT-IR and UV-Vis spectral redshift, and fluorescence quenching confirmed the formation of a new ground-state complex (Fig. 1F–I). This finding suggests that similar non-covalent interactions may exist between different components in TCM formulas, constituting the material basis for compatibility synergy.

Through chemical modification strategies [38], this study elucidated that carboxyl, hydroxyl, and carbonyl groups are all key functional groups for the SOD-like activity of LCDs. The SOD-like activity of BLCDs after BBR binding showed no significant difference from LCDs, indicating that BBR loading did not shield the active sites of LCDs (Fig. 2B and C). In LPS-induced cells, the intracellular antioxidant capacity of BLCDs was significantly superior to that of LCDs (Fig. 2G). This phenomenon is attributed to the indirect antioxidant effect of BBR within cells through multi-target regulation of anti-inflammatory pathways, forming a synergistic effect with the direct ROS scavenging by LCDs. BLCDs also demonstrated synergistic advantages in anti-inflammation, capable of restoring LPS-induced pro-inflammatory cytokine expression to near-normal levels (Fig. 2I and J).

Transcriptomic analysis revealed that IL10 was the most significantly upregulated gene after BLCDs treatment (Fig. 3A). Further validation showed that upon BLCDs upregulation of IL10, the expression of its downstream target molecule NLRP3 significantly decreased, subsequently inhibiting the secretion of effector pro-inflammatory cytokines IL1β and IL18 (Fig. S9B–D). IL10 inhibition experiments confirmed that blocking IL10 significantly weakened the anti-inflammatory and antioxidant effects of BLCDs, indicating that BLCDs primarily exert their therapeutic function by regulating IL10 (Fig. S12).

In the DSS-induced mice model, the therapeutic effect of BLCDs was superior to that of the positive drug 5-ASA and its individual components (Fig. 4). Colon length, pathology scores, goblet cell counts, and tight junction protein expression were all closest to those of the healthy group (Fig. 4F–I). Near-infrared imaging showed that after oral administration, BLCDs were primarily enriched in the intestine, with a retention time significantly longer than that of LCDs (Fig. 5I), attributable to the size-dependent retention effect conferred by their larger particle size [43,44]. In addition, given that UC is marked by negatively charged glycosylated components in colonic mucin [45], the conjugation of BBR with LCDs converted the surface charge of LCDs from negative to mildly positive (Fig. 1E), enabling BLCDs to interact with the negatively charged intestinal mucosa through electrostatic forces. This charge-based mechanism, which may also contribute to the prolonged retention.

16S rDNA sequencing revealed that BLCDs treatment significantly increased the abundance and diversity of the gut microbiota, promoted the proliferation of beneficial bacteria, and inhibited the growth of harmful bacteria such as *Klebsiella* (Fig. 6G). *Klebsiella* is involved in TMA metabolism [41,42], and its reduction was accompanied by a significant downregulation of TMAO levels in feces and plasma (Fig. 7B–D). CNO experiments confirmed that TMAO could directly induce inflammatory lesions, while BLCDs reversed this effect through IL10, forming a multi-level regulatory mechanism involving microbiota modulation to reduce TMAO production, which in turn activates IL10 (Fig. 7F and G, Fig. S25).

*In vitro* and *in vivo* safety assessments showed that BLCDs had no significant toxicity to normal cells or major mouse organs, with normal blood routine and liver/kidney function indicators (Fig. S20), suggesting good potential for clinical translation.

Several aspects of this study warrant further in-depth investigation. Whether the self-assembly behavior of BLCDs exists in real decoction systems and how upstream regulatory signals mediate the specific activation of STAT3/IL10 by BLCDs remain to be explored. Nevertheless, we have delineated their anti-inflammatory mechanisms. *In vitro*, BLCDs exhibited the most potent IL10 upregulation, primarily driven by the BBR component. Stattic-mediated inhibition confirmed that BLCDs activate p-STAT3 to upregulate IL10 and subsequently suppress NLRP3 (Fig. S11B). In DSS-induced mice, BLCDs similarly enhanced p-STAT3-mediated IL10 expression, successfully restoring DSS-elevated NLRP3 to baseline levels, highlighting the superior efficacy of this integrated formulation (Fig. S17). We proposed that BLCDs upregulated IL10 through a synergistic interplay. First, they decreased intracellular ROS to alleviate oxidative stress and activated p-STAT3. Second, the active component BBR broadly suppressesed pro-inflammatory mediators. Third, BLCDs inhibited *Klebsiella in vivo* to reduce TMAO production. Together, these actions disrupted the inflammatory microenvironment. Future studies integrating single-cell sequencing and spatial transcriptomics will further establish the theoretical basis for treating UC with BLCDs.

## 5 Conclusion

Drawing inspiration from the TCM formula Zanglian Wan, we developed a nano-assembly (BLCDs), formed through the self-assembly of *Sanguisorba officinalis L.*-derived carbon dots (LCDs) and BBR. This nano-assembly constructed a stable supramolecular structure through electrostatic interactions and hydrogen bonding, retaining both the SOD-like antioxidant activity of LCDs and the multi-target anti-inflammatory effects of BBR. The SOD-like activity was attributed to surface carboxyl, hydroxyl, and carbonyl groups. *In vitro* and *in vivo* experiments demonstrated that BLCDs exerted therapeutic effects through a dual synergistic mechanism. They directly scavenged ROS via surface oxygen-containing functional groups, while simultaneously inhibiting the NLRP3 pathway and downstream pro-inflammatory effector cytokines by p-STAT3-mediated IL10 expression. In DSS-induced mice, BLCDs exhibited superior efficacy compared to 5-ASA or either component alone, significantly ameliorating intestinal inflammation, oxidative stress, and barrier dysfunction. The therapeutic advantage stemmed from multiple factors, including enhanced intestinal retention due to larger particle size and charge reversal, and modulation of gut microbiota and metabolites, notably suppressing *Klebsiella* to reduce TMAO production, which in turn activated an IL10-mediated multi-level regulatory network. Safety assessments further confirmed the good biocompatibility of BLCDs, highlighting their potential for clinical translation. This study offers new insights into the compatibility mechanisms underlying TCM formulas and provides a reference paradigm for the development of nanomedicines based on active TCM components.

## CRediT authorship contribution statement

**Tao Luo:** Writing – original draft, Visualization, Validation, Funding acquisition, Data curation. **Meng Li:** Writing – review & editing, Data curation. **Ao Zhang:** Investigation. **Lu Wang:** Resources. **Qinguo Huang:** Data curation. **Zhen Wang:** Writing – review & editing, Supervision. **Kang Ding:** Supervision, Funding acquisition, Conceptualization.

## Ethics statement

This study complied with all the relevant national regulations and institutional policies, was in accordance with the Declaration of Helsinki and approved by the Ethics Committee of Nanjing Hospital of Chinese Medicine Affiliated to Nanjing University of Chinese Medicine (Approved Number: KY2022309). All animal experiment were performed in accordance with the protocols sanctioned by the Animal Ethical and Welfare (AEWC) at the Shenzhen Glorybay Biomedical Laboratory Animal Center (Approved Number: RW-IACUC-24-0131).

## Declaration of competing interests

The authors declare that they have no known competing financial interests or personal relationships that could have appeared to influence the work reported in this paper.

## Data Availability

Data will be made available on request.
