# Supplementary material for: Traditional Chinese Medicine-derived berberine-loaded herbzyme ameliorates ulcerative colitis by regulating IL10 and TMAO
Source: Mater Today Bio. 2026 Aug 27;40:103615. doi: 10.1016/j.mtbio.2026.103615 (PMC13553564; doi:10.1016/j.mtbio.2026.103615)
Supplement: Supplementary file 1 [file mmc1.docx]

**Supplementary Materials**

| **Table S1. Primer sequences used for qPCR** | | |
| --- | --- | --- |
| **Primer name** | **Forward primer sequence (5′-3′)** | **Reverse primer sequence (5′-3′)** |
| IL6 | AGACAGCCACTCACCTCTTCAG | TTCTGCCAGTGCCTCTTTGCTG |
| IL10 | TCTCCGAGATGCCTTCAGCAGA | TCAGACAAGGCTTGGCAACCCA |
| NLRP3 | GGACTGAAGCACCTGTTGTGCA | TCCTGAGTCTCCCAAGGCATTC |
| IL1β | CCACAGACCTTCCAGGAGAATG | GTGCAGTTCAGTGATCGTACAGG |
| IL1ɑ | TGTATGTGACTGCCCAAGATGAAG | AGAGGAGGTTGGTCTCACTACC |
| Caspase-1 | GGCACATTTCCAGGACTGACTG | GCAAGACGTGTACGAGTGGTTG |
| FMO3 | GGAAAACGAGTCCTGGTGATTGG | CGACTCATCACCCAAGAACCAC |
| TNFɑ | CTCTTCTGCCTGCTGCACTTTG | ATGGGCTACAGGCTTGTCACTC |
| GAPDH | GTCTCCTCTGACTTCAACAGCG | ACCACCCTGTTGCTGTAGCCAA |

| **Table S2. Thermodynamic parameters for the binding of BBR to LCDs determined by ITC at 298 K** | | | | | | |
| --- | --- | --- | --- | --- | --- | --- |
| Condition | Environment | K_D_ (μM) | ΔH (kcal/mol) | TΔS (kcal/mol) | ΔS (cal/mol·K) | ΔG (kcal/mol) |
| Reference | pH 7.4, 10 mM PBS | 2.18 ± 0.19 | 1.92 ± 0.14 | 9.64 | 32.33 ± 1.15 | -7.72 ± 0.16 |
| Acidic pH | pH 5.5, 10 mM PBS | 9.64 ± 0.61 | 2.31 ± 0.17 | 9.15 | 30.69 ± 0.98 | -6.84 ± 0.20 |
| High Salt | pH 7.4, 150 mM NaCl | 18.53 ± 1.42 | -3.18 ± 0.21 | 3.28 | 11.00 ± 0.74 | -6.46 ± 0.18 |
| Serum Protein | pH 7.4, 10 mg/mL BSA | 21.80 ± 1.68 | -2.95 ± 0.19 | 3.41 | 11.44 ± 0.87 | -6.36 ± 0.17 |
| H-bond Disruptor | pH 7.4, 150 mM NaCl + 2M Urea | 85.40 ± 4.25 | -1.15 ± 0.10 | +4.40 | 14.75 ± 1.22 | -5.55 ± 0.25 |

**Figure S1.** High‑resolution TEM (HRTEM) image of LCDs. The regions of magnified lattice fringes are enclosed by blue dashed circles, with lattice fringes indicated by white arrows.

**Figure S2.** (A) SAED pattern of LCDs; the inset in the lower right corner indicates the TEM measurement area. (B) XRD pattern of LCDs. (C) Raman spectroscopy data of LCDs. (D) Loading efficiency of BBR in complexes formed with LCDs at different mass ratios (n = 3).

**Figure S3.** Hydrodynamic diameter and PDI of LCDs after 7 days of incubation in various dispersion media, including ddH_2_O, PBS, and simulated intestinal fluid (SIF).

**Figure S4.** (A) FT‑IR spectra of LCDs, LCDs‑PS, and LCDs‑PS‑HCl. XPS C 1s high‑resolution spectra of (B) LCDs‑NaBH_4_ and (C) LCDs‑NaBH_4_‑HNO_3_.

**Figure S5.** Cell viability of NCM460 cells treated with different concentrations of (A) LCDs, and (B) BLCDs (n = 3).

**Figure S6.** Cell viability was assessed using CCK‑8 assay. NCM460 cells were stimulated with LPS and then treated with various concentrations of BBR, LCDs, or BLCDs (0, 1.5625, 3.125, 6.25, 12.5, 25, 50, and 100 μg/mL) for 48 h.

**Figure S7.** (A) Release of BBR from BLCDs into the dialysate under three simulated gastrointestinal conditions: simulated gastric fluid (SGF, pH 1.2, containing NaCl and pepsin), simulated intestinal fluid (SIF, pH 6.8, containing KH_2_PO_4_ and pancreatin), and simulated colonic fluid (SCF, pH 7.4, prepared as PBS). (B) Cellular uptake of BBR at different incubation times (24, 48, and 72 h) in cells treated with BBR (33.3 μg/mL), BLCDs (50 μg/mL), or a physical mixture of BBR (33.3 μg/mL) and LCDs (16.7 μg/mL).

**Figure S8.** Dose‑response curves of LPS‑stimulated NCM460 cells treated with graded concentrations (1.5625, 3.125, 6.25, 12.5, 25, 50, and 100 μg/mL) of BBR, LCDs, or BLCDs. The combination index (CI) was calculated using CompuSyn software based on the fraction affected (Fa) values derived from the median‑effect principle, where CI < 1, CI = 1, and CI > 1 indicate synergism, additivity, and antagonism, respectively.

**Figure S9.** (A) Radar chart showing transcriptomic changes in IL10-related pathway genes before and after BLCDs treatment. (B) mRNA and protein expression levels of IL10 (n = 3). (C) mRNA and protein expression levels of NLRP3 (n = 3). (D) Expression levels of IL1β and IL18 (n = 3). (E) Expression levels of TNFα and IL6 (n = 3).

**Figure S10.** IL10 levels (measured by ELISA) in culture supernatants of NCM460 cells under indicated treatments: Con, LPS-stimulated cells; LCDs, LPS+LCDs (16.7 μg/mL, 48 h); BBR, LPS+BBR (33.3 μg/mL, 48 h); BBR+LCDs, LPS+physical mixture of BBR (33.3 μg/mL) and LCDs (16.7 μg/mL, 48 h); BLCDs, LPS+BLCDs (50 μg/mL, 48 h).

**Figure S11.** (A) Ratios of OD450 values for total transcription factors (NF-ĸB p65, c-Jun, C/EBP-β, and STAT3) and their phosphorylated/activated forms (p-p65, p-c-Jun, p-C/EBP-β, and p-STAT3) in LPS-stimulated NCM460 cells treated with BLCD, relative to the control (LPS-stimulated cells without treatment). (B) Western Blot analysis of STAT3, p-STAT3, IL10, NLRP3, and GAPDH in NCM460 cells under different treatment conditions. PBS, cell only treated with PBS; LPS, LPS-stimulated cells; LCDs, LPS-stimulated cells treated with LCDs (16.7 μg/mL, 48 h); BBR, BBR (33.3 μg/mL, 48 h); BBR+LCDs, LPS-stimulated cells treated with physical mixture of BBR (33.3 μg/mL) and LCDs (16.7 μg/mL, 48 h); BLCDs, LPS-stimulated cells treated with BLCDs (50 μg/mL, 48 h); BLCDs+Stattic, LPS-stimulated cells co-treated with BLCDs (50 μg/mL, 48 h) and Stattic (10 μM, 48 h). The primary antibodies used were as follows: anti‑STAT3 (abcam, ab68153, 1:2000), anti‑p‑STAT3 (abcam, ab76315, 1:5000), anti‑IL‑10 (Invitrogen, PA5‑85660, 1:5000), anti‑NLRP3 (abcam, ab263899, 1:1000)), and anti‑GAPDH (abcam, ab181602, 1:10,000). Primary antibodies were incubated overnight at 4°C. The secondary antibody, goat anti‑rabbit IgG H&L (HRP) (abcam, ab6721), was applied at a dilution of 1:5000 and incubated for 1 h at room temperature.

**Figure S12.** RAW264.7 cells under different treatment conditions, showing (A) Expression level of IL10 (n = 3); (B) Expression level of NLRP3 (n = 3); (C) Expression levels of IL1β and IL18 (n = 3); (D) Expression levels of TNFα and IL6 (n = 3); (E) ROS fluorescence intensity (n = 3); (F) Cell viability (n = 3). JES5-2A5 is an IL10 monoclonal antibody used at a final concentration of 5 μg/mL, co-incubated with BLCDs (50 μg/mL) for 48 h.

**Figure S13.** IL10 expression levels in NCM460 or RAW264.7 cells treated with various concentrations of BLCDs, with or without LPS stimulation.

**Figure S14.** Intracellular levels of (A) MDA and (B) GSH in NCM460 cells following various treatments. PBS, cell only treated with PBS; LPS, LPS-stimulated cells; LCDs, LPS-stimulated cells treated with LCDs (16.7 μg/mL, 48 h); BBR, BBR (33.3 μg/mL, 48 h); BBR+LCDs, LPS-stimulated cells treated with physical mixture of BBR (33.3 μg/mL) and LCDs (16.7 μg/mL, 48 h); BLCDs, LPS-stimulated cells treated with BLCDs (50 μg/mL, 48 h).

**Figure S15.** Effect of different concentrations of BLCDs on the DAI score in DSS-induced mice (n = 6).

**Figure S16.** Perianal contamination scores based on the percentage of perianal area covered by feces. Scores were defined as follows: 0, normal (0% soiling); 1, mild (1–10% soiling); 2, moderate (11–30% soiling); 3, severe (>30% soiling).

**Figure S17.** Western Blot analysis of STAT3, p-STAT3, IL10, NLRP3, Occludin, ZO-1, and GAPDH in colon tissue samples. The primary antibodies used were as follows: anti‑STAT3 (abcam, ab68153, 1:2000), anti‑p‑STAT3 (abcam, ab76315, 1:5000), anti‑IL‑10 (Invitrogen, PA5‑85660, 1:5000), anti‑NLRP3 (abcam, ab263899, 1:1000), anti‑occludin (abcam, ab216327, 1:1000), anti‑ZO‑1 (abcam, ab307799, 1:1000), and anti‑GAPDH (abcam, ab181602, 1:10,000). Primary antibodies were incubated overnight at 4°C. The secondary antibody, goat anti‑rabbit IgG H&L (HRP) (abcam, ab6721), was applied at a dilution of 1:5000 and incubated for 1 h at room temperature.

**Figure S18.** H&E staining images of major tissues (heart, liver, spleen, lungs, kidneys).

**Figure S19.** (A) *Caspase‑1* mRNA expression in colon tissue samples from different groups. (B) *FMO3* mRNA expression in liver tissue samples from different groups.

**Figure S20.** (A) Schematic diagram of the animal safety experiment. (B) Body weight changes in mice (n = 10). (C) H&E staining images of major tissues. (D) Hematological parameters of mice (n = 5). (E) Liver and kidney function indicators in mice (n = 5). (F) Hemolysis rate of BLCDs (n = 3). (G) Metabolic data of BLCDs in colon tissues at 0.167, 0.5, 1, 2, 4, 6, 8, 12, 24, 48, and 72 h.

**Figure S21.** (A) Principal component analysis (PCA) score plot. (B) Heatmap of gut metabolomics among groups (PBS, DSS, 5-ASA, LCDs, BBR, BBR+LCDs, BLCDs). Color bar (based on log_2_ fold change) indicate relative levels.

**Figure S22.** TMA synthesis-related genes, (A) cutC and (B) cutD, in *Klebsiella*. (C) Schematic representation of the experimental protocol for *Klebsiella* (ATCC 43816) colonization and treatment in germ-free mice (n = 6). (D) *Klebsiella* colony counts (colony-forming units, CFU) per gram of colon tissue. (E) Quantification of cutC and cutD levels. (F) Fecal TMA and (H) TMAO concentrations across the experimental groups. Germ-free group: treated with PBS only; *Klebsiella* group: orally administered with 2×10^6^ CFU of ATCC 43816 (p.o.); *Klebsiella*+BLCDs group: treated with BLCDs (80 mg/kg, p.o.) in *Klebsiella*‑colonized mice; *Klebsiella*+DMB group: positive control, treated with DMB (200 mg/kg, p.o.) in *Klebsiella*‑colonized mice.

**Figure S23.** (A) The diameter of CNOs (n = 3). (B) The viability of CNOs (n = 3). (C) MDA level in the culture medium of CNOs (n = 3).

**Figure S24.** Histopathological scoring of H&E‑stained sections of CNOs.

**Figure S25.** (A) Immunohistochemical results for IL10 in CNOs under various treatments. (B) Expression levels of IL10 (n = 3). (C) Bright-field images, (D) The diameter of CNOs (n = 3). (E) The viability of CNOs (n = 3). (F) MDA level in the culture medium of CNOs (n = 3).
